# Single-Cell Sequencing Reveals Circadian Sensitivity of Noise-Induced Hearing Loss Mediated by Macrophage-Driven NLRP3 Inflammasome Activation

**DOI:** 10.1007/s12264-025-01440-1

**Published:** 2025-07-20

**Authors:** Qingping Ma, Qixuan Wang, Zixuan Zhu, Qian Zhou, Zhongying Wang, Minfei Qian, Teng Li, Xixi Gu, Zechuan Chen, Xueling Wang, Xiaoming Zhang, Zhiwu Huang

**Affiliations:** 1 Department of Otolaryngology-Head and Neck Surgery, Shanghai Ninth People’s Hospital, Shanghai Jiao Tong University School of Medicine, Shanghai, 200011 China; 2 Ear Institute, Shanghai Jiao Tong University School of Medicine, Shanghai, 200125 China; 3 Shanghai Key Laboratory of Translational Medicine on Ear and Nose Diseases, Shanghai, 200125 China; 4 Department of Facial Plastic and Reconstructive Surgery, Eye & ENT Hospital of Fudan University, ENT Institute, Eye & ENT Hospital of Fudan University, NHC Key Laboratory of Hearing Medicine, Fudan University, Shanghai, 200031 China; 5 National Key Laboratory of Immune Response and Immunotherapy, Shanghai Institute of Immunity and Infection, Chinese Academy of Sciences, Shanghai, 200031 China; 6 Department of Otolaryngology-Head and Neck Surgery, Renji Hospital, Shanghai Jiao Tong University School of Medicine, Shanghai, 200127 China; 7 Biobank, Shanghai Ninth People’s Hospital, Shanghai Jiao Tong University School of Medicine, Shanghai, 200125 China; 8 College of Health Science and Technology, Shanghai Jiao Tong University School of Medicine, Shanghai, 200025 China

**Keywords:** Noise-induced hearing loss, Macrophage, NLRP3 inflammasome, Circadian rhythm

## Abstract

Circadian sensitivity significantly influences the severity of noise-induced hearing loss (NIHL), but the underlying mechanisms remain unclear. Here, we applied single-cell RNA sequencing to 97,043 cochlear cells, identifying macrophages as the primary immune responders to acoustic trauma, with a notable increase in their proportion in the cochlea. Immunofluorescence confirmed significant recruitment and activation of cochlear macrophages following noise exposure, while *in vivo* macrophage depletion resulted in the recovery of hearing. Furthermore, analyses of differentially-expressed genes and pathways revealed pronounced activation of NLRP3 inflammasome signaling in macrophages during night-time noise exposure. Measurements of elevated IL-1β and IL-18 expression in cochlear macrophages by multiplex immunohistochemistry correlated with heightened inflammation in the night-time exposure group. These findings were further confirmed by the administration of the selective NLRP3 inhibitor CY-09, which mitigated inflammasome activation, preserved synaptic integrity, and protect against hearing loss. In conclusion, our findings underscore the role of macrophage-driven NLRP3 inflammasome activation in mediating circadian variations in cochlear damage, offering a potential therapeutic target for mitigating NIHL.

**Supplementary Information:**

The online version contains supplementary material available at 10.1007/s12264-025-01440-1.

## Introduction

Noise-induced hearing loss (NIHL) is one of the most prevalent causes of acquired sensorineural hearing loss, affecting over 1 billion individuals aged 12–35 due to prolonged exposure to loud noise, according to the World Health Organization [1]. Beyond impairing auditory perception, NIHL contributes to broader public health challenges, including cognitive decline and neurodegenerative conditions such as dementia and presbycusis [2, 3]. Despite its profound social and economic implications, effective prevention and therapeutic strategies for NIHL remain elusive.

While significant strides have been made in understanding the pathophysiology of NIHL, key mechanisms underlying its development remain unresolved. Emerging evidence highlights the circadian rhythm as a critical factor influencing NIHL susceptibility. Cohort studies of noise-exposed populations have shown an increased risk of hearing loss among shift workers [4, 5], implicating temporal variability in susceptibility to auditory damage. Complementing these findings, animal studies have revealed that cochlear sensitivity to acoustic trauma varies with circadian timing. For instance, Melster *et al.* demonstrated that male CBA/CaJ mice exposed to identical noise conditions at 9 a.m. and 9 p.m. experienced significantly greater permanent threshold shifts at night [6]. These studies highlight the circadian sensitivity of NIHL but fall short of explaining the cellular and molecular mechanisms driving this phenomenon, thereby hindering the development of targeted interventions. Investigating circadian-specific mechanisms offers a novel approach to uncovering NIHL pathophysiology and could provide transformative insights for both preventive and therapeutic strategies.

Traditionally, research on NIHL has centered on mechanical damage [7–11] and the subsequent metabolic stress [12–15]. More recently, immune and inflammatory responses have emerged as pivotal modulators of noise-induced cochlear damage [16, 17]. The cochlea is now recognized as an immune-active environment, with mononuclear phagocytes, particularly macrophages, playing critical roles in mediating immune responses to noise exposure [18, 19]. However, it remains unclear how macrophage activation and inflammatory dynamics contribute to circadian differences in NIHL susceptibility. Furthermore, the immune system itself is influenced by circadian rhythms [20, 21], raising the possibility that circadian modulation of immune responses shapes the temporal variability in NIHL.

Single-cell RNA sequencing (scRNA-seq) has emerged as a transformative technology for dissecting cellular heterogeneity and understanding immune responses in disease development or therapeutic interventions. Applying scRNA-seq to cochlear tissues presents unique challenges due to the small size, delicate structure, and limited number of cochlear cells, as well as their short *ex vivo* viability. Despite these challenges, scRNA-seq offers an unprecedented opportunity to comprehensively profile the cochlear immune landscape, particularly under conditions of stress such as noise exposure. While pioneering studies have identified immune cells in the cochlea at single-cell resolution [13, 22, 23], a systematic investigation of immune dynamics, especially macrophage-driven inflammatory responses, remains lacking.

Here, we utilized scRNA-seq to characterize cochlear cells before and after noise exposure, focusing on circadian-specific immune dynamics. Our analysis identified macrophages as the primary immune cell population responding to acoustic trauma, characterized by heightened NLRP3 inflammasome activation, particularly during night-time noise exposure (NNE). Functional experiments demonstrated that targeting the NLRP3 inflammasome with the selective inhibitor CY-09 effectively reduced inflammation and alleviated auditory damage. By uncovering the macrophage-driven inflammatory pathways underlying the circadian sensitivity of NIHL, our findings provide critical insights into how temporal dynamics modulate cochlear immune responses and susceptibility to noise-induced damage. These findings highlight the importance of circadian regulation in shaping NIHL susceptibility and provide a framework for exploring time-sensitive therapeutic strategies targeting macrophage-mediated inflammation to mitigate NIHL.

## Materials and Methods

### Animal Maintenance

Male CBA/CaJShjh mice, aged 6–8 weeks (obtained from Shanghai Jihui Laboratory Animal Breeding Co., Ltd, China), were provided with adequate food and water and housed in standard cages. All mice were maintained under a 12-h light/12-h dark cycle. Mice of the same age were randomly assigned to each experimental group. Our study was confined to male mice due to documented sex differences in NIHL, with males exhibiting greater susceptibility [24]. This choice aimed to reduce the variability and facilitate a focused investigation of circadian sensitivity to NIHL. The applicability of these findings to female mice remains to be determined.

### Noise Exposure Paradigm

Noise exposure was induced by exposing awake and unanesthetized animals to a calibrated reverberation chamber, where sound pressure level variations were ~1 dB across typical locations. Bandpass noise (2–20 kHz) at 107 dB sound pressure level (SPL) was delivered through an amplifier and loudspeaker (Yamaha, PX10, Japan) for 2 h. The SPL was calibrated to the target level before each acoustic exposure using an acoustic meter (Hangzhou Aihua, AWA6228+, China), as previously described [25]. Zeitgeber time (ZT) 0 corresponds to the onset of daylight (6 a.m.), while ZT 12 corresponds to the onset of night-time (6 p.m.). Experiments were performed on the daytime noise exposure (DNE) group at ZT 2–4 and the NNE group at ZT 14–16.

### Auditory Brainstem Response (ABR) Recordings

ABRs were recorded in a sound-attenuating chamber with background noise levels <30 dB(A), using an electrophysiology workstation (Tucker-Davis Technologies, RZ6, USA). ABRs were recorded before noise exposure and 1 day and 14 days post-exposure. All animals were anesthetized using zoletil (50 mg/kg) and xylazine (20 mg/kg) by intraperitoneal injection, and body temperature was maintained at 37°C with an isothermal pad (Harvard Apparatus, 55-7020, USA). Subdermal needle electrodes were placed at the vertex of the skull (active), the mastoid area of the left ear (reference), and the right shoulder (ground). Free-field sound stimuli were delivered through a speaker (Tucker-Davis Technologies, MF1, USA) positioned 10 cm from the vertex. For consistency, all ABR recordings in response to acoustic stimulation (4000, 8000, 11314, 16000, 22627, and 32000 Hz) were made by the same experimenter. The stimulus started at 90 dB SPL and decreased in 5 dB SPL steps until one level below visible responses. Each waveform was averaged 400 times. Thresholds were defined as the lowest stimulus level at which a response was recorded, with the response amplitude being more than twice the height of the noise. Latencies and amplitudes of ABR wave I were measured using BioSigRZ software. Latency was defined as the time between signal onset and the peak, while amplitude was calculated by averaging ΔV of both sides of wave I [26].

### Distortion Product Otoacoustic Emission (DPOAE) Recordings

DPOAEs arise from the nonlinearity of the cochlear response when stimulated at two closely-spaced frequencies, f1 and f2. The interaction produces sounds known as intermodulation distortion products (DPs), with the most intense occurring at a frequency of 2f1−f2. DPOAEs were measured before and 14 days after DNE and NNE, as described in our previous study [25]. The two stimulus frequencies (f1 and f2) were generated by an electrophysiology workstation (Tucker-Davis Technologies, RZ6, USA) driving two MF1 speakers, which delivered equal-intensity primary tones with a frequency ratio (f2/f1) of 1.2. The speakers were connected to the ear canal in a closed-field configuration. Stimuli ranged from 80 dB to 20 dB SPL, with f1 and f2 intensities decreased in steps of 5 dB SPL, targeting frequencies at 8000, 16000, and 32000 Hz. The amplitudes of DPs at the frequency 2f1−f2 were averaged 512 times. The DPOAE threshold was defined as the point at which DP can no longer be distinguished from the noise [27].

### Scanning Electron Microscopy (SEM)

Mice were anesthetized and perfused intracardially with fixative (2.5% glutaraldehyde and 2% paraformaldehyde (PFA)). We collected cochleae immediately after cardiac perfusion and removed a piece of bone from the apical end of each cochlea. Fixative was gently injected through the round and oval windows, and the cochleae were incubated in 2.5% glutaraldehyde overnight at 4°C, followed by decalcification in 10% ethylene diamine tetraacetic acid (EDTA) at room temperature (RT) until completed. After removing the tectorial membrane, the organ of Corti was separated and post-fixed in 1% OsO_4_ for 1 h at 4°C. Specimens were then fixed in 1% thiocarbohydrazide for 30 min at RT, followed by another fixation in 1% OsO_4_ for 1 h at 4°C. The cochleae were subsequently dehydrated through a series of graded ethanol incubations, dried at the critical point, and mounted on stubs [28]. After sputter-coating with gold in a vacuum coater (Leica, EM ACE200, Germany), samples were characterized under a field emission scanning electron microscope (Zeiss, GeminiSEM 300, Germany) using a secondary electron detector.

### Whole-Mount Staining of Cochleae, Confocal Imaging, and Analysis

Immediately after the mice were sacrificed, the temporal bones were removed and the cochleae of both ears were collected. 4% PFA solution was gently injected through the round and oval windows, and the cochleae were stored in the same solution overnight at 4°C. The 4% PFA was then replaced with 10% EDTA until decalcification was complete. The cochleae were dissected into the three turns and blocked with a solution containing 0.3% Triton, 5% bovine serum, and 5% donkey serum in 0.01 mol/L phosphate buffered saline (PBS) for 1 h at RT. The tissues were incubated overnight at 4°C with primary antibodies in blocking solution: rabbit polyclonal anti-Myosin VIIa (1:200, Proteus BioSciences, 25–6790, USA), mouse (IgG1) anti-CtBP2 (1:200, BD Biosciences, 612044, USA), mouse (IgG2a) anti-Glutamate Receptor (1:50, BD Biosciences, 556341, USA), goat polyclonal IgG anti-CD45 (1:100, R&D systems, AF114, USA), and rat (IgG2a) anti-CD68 (1:100, Bio-Rad, MCA1957GA, USA).

Following overnight incubation with primary antibodies at 4°C, the tissues were washed three times with 0.01 mol/L PBS and incubated with the species-specific secondary antibodies Alexa Fluor 488-conjugated goat anti-rabbit IgG (H+L) (Invitrogen, A-11008, USA), Alexa Fluor 633-conjugated goat anti-mouse IgG1 (Invitrogen, A-21126, USA), Alexa Fluor 555-conjugated goat anti-mouse IgG2a (Invitrogen, A-21137, USA), Alexa Fluor 488-conjugated donkey anti-goat IgG (H+L) (Yeasen Biotech, 34306ES60, China), and Alexa Fluor 568-conjugated goat anti-rat IgG (H+L) (Invitrogen, A-11077, USA) for 1 h at RT. After washing three times in 0.01 mol/L PBS, samples were mounted with DAPI Fluoromount-G (SouthernBiotech, 0100-20, USA) on clean glass slides.

Confocal images were acquired using a confocal laser scanning microscope (Carl Zeiss, Zeiss LSM 880, Germany) in z-stack mode and preprocessed to show the maximum intensity projection. Hair cells labeled with Myosin VIIa and macrophages in the basilar membrane (BM) and osseous spiral lamina (OSL) were examined and counted with a 20× objective lens. To quantify the number of ribbon synapses per inner hair cell (IHC), presynaptic CtBP2 puncta that overlapped with postsynaptic GluR2 puncta were considered a single synaptic unit, counted under a 63× oil immersion lens.

### Single-Cell Isolation and Sequencing

The organ of Corti, along with the lateral wall and modiolus containing the spiral ganglion, was isolated and dissociated into a single-cell suspension using a Dissociation Kit (Miltenyi Biotec, 130-096-730, Germany). Mouse cochlear cells were isolated in cold Dulbecco's PBS without Ca^2+^ and Mg^2+^, and 1% fetal bovine serum (Gibco, A3161001C, USA) was added to generate single-cell suspensions. Cells were then filtered through a 40-μm cell-strainer nylon mesh (Falcon, 352235, USA). Live cells (DAPI-negative, DRAQ5-positive) were sorted using a FACS Aria II (BD Biosciences, USA) and subsequently subjected to single-cell analysis. The sorted cells were sequenced using the 10× Chromium single-cell platform with 3' Reagent Kits, according to the manufacturer's protocol. The single-cell libraries were sequenced using the Illumina NovaSeq platform.

### ScRNA-seq Data Quality Control, Processing, Annotation, and Visualization

The dataset was aligned and quantified using the CellRanger single-cell software suite (V3.1.0) with default settings. The CellRanger count function was used to quantify the sample-specific FASTQ file and aligned to the mouse reference genome (mm10). For downstream analysis, the filtered gene expression matrix was used.

The downstream analysis of scRNA-seq data was conducted using the single-cell toolkit Seurat (V5.1.0) [29] in R (V4.1.2). To ensure high-quality cells, only those with at least 500 detected genes and fewer than 10% mitochondrial genes were included. To minimize false positives related to biological heterogeneity and technical factors such as sequencing depth, feature counts were normalized using the LogNormalize function in Seurat [29] with a scaling factor of 10,000. For variable gene selection, we identified features with the highest coefficients of variation using the FindVariableFeatures function in Seurat [29], selecting the top 2,000 variable features for dimensionality reduction by default. To address batch effects, the principal component analysis matrix containing 40 components was transferred to Harmony (V0.1.1) [30]. In the batch-corrected space, uniform manifold approximation and projection (UMAP) implemented through the RunUMAP function in Seurat [29], was applied for dimensionality reduction, with visualization based on the resulting cell embedding coordinates.

We conducted systematic two-round unsupervised clustering using the FindClusters function in Seurat [29], setting the resolution at 1.0 and utilizing the top 30 principal components to define cell identities. In the first round of clustering, cells were divided into hematopoietic (*Ptprc*^+^, *Hbb*^+^) and non-hematopoietic types due to the presence of a hematopoietic system in the cochlea. Clusters exhibiting high expression of two or more lineage markers simultaneously were identified as putative doublets and excluded from further analysis [31]. The identification of major cell types and the removal of doublets were conducted iteratively to ensure the purity of all primary cellular compartments. An additional round of unsupervised clustering was then allied to identify fine-grained cell subtypes using the previously described methods. Marker genes for each cluster were identified using the Wilcoxon rank-sum test through the FindAllMarkers function in Seurat [29]. Only genes with |avg_logFC| >0.5, min.pct >0.5, and *P*_adj_ < 0.05 were considered marker genes. Cell types were identified based on the expression of established marker genes, with the specific differentially-expressed genes (DEGs) for each cell type listed in Tables S1 and S2.

We applied cluster analysis using TooManyCells (V2.2.0.0) [32] to visualize the relationships among cell clades and identify significant differences between the control and noise-exposed groups. The cell clades facilitated the visualization of differences among cell subsets, with the background region specifically highlighting the activation of cochlear macrophages following noise exposure. To quantify cell type enrichment across groups, we calculated the observed *versus* expected cell numbers in each cluster using the formula Ro/e = Observed/Expected [33], where the expected cell numbers for each cell cluster are derived from the χ^2^ test. A cluster was considered enriched in a particular group if the Ro/e ratio was >1.

### Functional Enrichment, Differential Gene Expression, and SCENIC Analysis

To assess pathway activity at the single-cell level, we used the VISION package (V3.0.1). Canonical pathway gene sets were obtained from the MsigDB database (V2023.2) [34, 35] and used to score individual cells. We utilized the FindMarkers function in Seurat [29] to identify noise-associated DEGs between the control group and the DNE or NNE group in non-immune cells. Only genes with |avg_logFC| >0.25 and *P*_adj_ < 0.05 were classified as noise-associated DEGs (specific DEGs for each non-immune cell type are detailed in Table S3). To further annotate the functional implications of these DEGs, we applied Gene Ontology (GO) term enrichment analysis using ClusterProfiler (V4.2.2), with default settings (datasets: org.Mm.eg.db V3.18.0).

We applied single-cell regulatory network inference and clustering (SCENIC) analysis [36] to predict the transcriptional regulatory network in macrophages, using the 10,000 motifs database from RcisTarget (V1.14.0), along with GENIE3 (V0.16.0) and AUCell (V1.16.0). The normalized expression matrix generated by Seurat served as the input. After processing, the resulting matrix of transcription factor AUCell scores was integrated into the Seurat S4 object. Due to layout constraints, only 25 regulated targets per transcription factor were sampled and visualized using Igraph (V1.5.1).

### Analysis of Ligand-Receptor Interaction

We used CellChat (V1.6.1) [37] to analyze ligand-receptor interactions between immune and non-immune cells using default settings. For each cell group, both outgoing and incoming interaction strengths were calculated. In addition, we used CellPhoneDB (V5.0) [38] to examine the ligand-receptor interaction between non-immune cells and immune cells, particularly macrophage clusters. Since the CellPhoneDB database includes both “ligand-receptor” and “receptor-ligand” notations for interactions, all interactions were standardized to consistently follow the ligand-receptor format.

### Inner Ear Total RNA Extraction and Bulk RNA Sequencing

Cochleae were collected as described previously. The tissue was immediately transferred to a clean dish containing ice-cold RNAlater solution (Invitrogen, AM7024, USA). Total RNA was extracted from the excised tissue using the RNAiso Plus (Takara, 9108, Japan) following the manufacturer’s protocol. The purity and concentration of RNA were assessed using a NanoDrop 2000 spectrophotometer (Thermo Scientific, USA). The integrity and quality of the RNA were assessed using the Agilent 2100 Bioanalyzer (Agilent Technologies, USA). Libraries were then constructed using the mRNA Library Prep Kit (Yeasen Biotech, China) according to the manufacturer’s instructions. These libraries were sequenced on an Illumina HiSeq X Ten platform at Berry Genomics (Beijing, China), generating 150-bp paired-end reads. The raw reads in FASTQ format were initially processed using fastp (V0.23.4) [39], where low-quality reads were removed to obtain clean reads. The clean reads were subsequently mapped to the mouse reference genome (mm10) using the STAR (V2.7.10b) alignment tool [40]. The fragments per kilobase of transcript per million mapped reads [41] for each gene were calculated, and the read counts of each gene were determined using FeatureCounts (V2.0.6) [42].

### Western Blotting Analysis

Total cochlear protein was extracted using RIPA lysis buffer containing 1% protease inhibitor cocktail (Roche Diagnostics, 11873580001, Germany) and 1% phosphatase inhibitor cocktail (Thermo Fisher Scientific, WG332461, USA), following the manufacturer’s instructions. Protein was quantified using the BCA protein assay kit (Beyotime Biotech, P0012S, China). Protein extracts were subjected to SDS-PAGE and transferred to PVDF membranes (Millipore, IPVH00010, and Immobilon-P, USA). After blocking with 5% non-fat dried milk for 1 h, the protein bands were incubated with primary antibodies at 4°C overnight. The following primary antibodies were used: rabbit anti-NLRP3 (Cell Signaling Technology, 15101, USA), rabbit anti-phospho-NF-κB p65 (Cell Signaling Technology, 3033, USA), rabbit anti-IL-1 beta (Abcam, ab234437, USA), rabbit anti-IL-18 (Abcam, ab207323, USA), rabbit anti-pro-caspase-1 (Abcam, ab179515, USA), and mouse anti-β-Actin antibody (Beyotime, AF0003, China). The next day, the membranes were washed with TBST (20 mmol/L Tris–HCl, 500 mmol/L NaCl, and 0.1% Tween-20) and then incubated with horseradish peroxidase (HRP)-conjugated secondary antibodies for 1 h. After washing three times, protein bands were visualized using an enhanced chemiluminescence kit (Meilunbio, MA0186, China) on an Amersham Imager 600 (GE Healthcare, USA). The images were quantified using ImageJ software (National Institutes of Health, USA).

### Multiplex Immunohistochemical (mIHC) Staining and Imaging of the Cochlea

The fixation and decalcification processes were the same as those used for whole-mount staining of the cochlea described above. The cochleae were dehydrated through a series of graded ethanol incubations, then embedded in paraffin, and sectioned at 5–7 μm along the mid-modiolar axis. The sections were deparaffinized in xylene and rehydrated in a graded ethanol series. For antigen retrieval, an EDTA (pH 9.0) solution was preheated to 95°C for 15 min, and samples were stored until they cooled to RT. Endogenous peroxidase activity was blocked by incubating the samples in 3% H_2_O_2_ in 0.01 mol/L PBS for 20 min, followed by blocking with goat serum (Vector, S-1012-50, USA) for an additional blocking step.

Slides were then incubated with a primary antibody solution (1:1000 dilution) for 1 h at RT, labeled with HRP-conjugated secondary antibody for 20 min at RT, and visualized using tyramide signal amplification (TSA) fluorescent dyes (Panovue, 10001100100, China). Sections were prepared as previously described for additional antibodies, which required heating in citric acid buffer (pH 6.0) and sequential incubation with goat serum, primary antibodies, secondary antibodies, and TSA fluorescent dyes. The following primary and secondary antibodies were used: recombinant rabbit monoclonal anti-CD68 (Abcam, ab303565, USA), recombinant rabbit monoclonal anti-IL-18 (Abcam, ab223293, USA), recombinant rabbit monoclonal anti-IL-1 beta (Abcam, ab234437, USA), and the ImmPRESS® HRP Goat Anti-Rabbit IgG Polymer Detection Kit (Vector, MP-7451, USA). Following nucleus staining with DAPI, slides were scanned and images were acquired using a confocal laser scanning microscope (Carl Zeiss, Zeiss LSM 880, Germany).

### Drug Treatments

Clodronate liposomes (CLs) (Yeasen Biotech, 40337ES08, China) were administered to mice *via* caudal vein injection at a dose of 200 µL per mouse. Injections were given once before noise exposure and again one day after. CY-09 (5 mg/kg, MedChemExpress, HY-103666, USA), an NLRP3 inhibitor with excellent selectivity and specificity [43], or a vehicle solution (5% DMSO, 40% PEG300, 5% Tween 80, and 50% saline) was administered intraperitoneally once daily for 10 consecutive treatments. Noise exposure was initiated 7 days after drug administration.

### Statistical Analysis

We defined a statistically significant difference as *P* < 0.05. All sequencing data were assessed using R (V4.1.2) and experimental data were analyzed and plotted using GraphPad Prism (V8.0) software. For comparisons between two groups, unpaired two-tailed Student's *t*-tests were applied. For comparisons involving more than two groups, one-way or two-way analysis of variance (ANOVA) with Bonferroni's correction for multiple comparisons was utilized. Unless otherwise stated, bar plots are presented as the mean ± standard deviation (SD; error bar). All experiments were conducted in triplicate or greater and independently repeated.

### Ethics Statement

All animal experiments were approved by and performed in accordance with the guidelines of the Institutional Animal Care and Use Committee of the Ninth People’s Hospital, Shanghai Jiao Tong University School of Medicine (approval number SH9H-2020-A712-1).

## Results

### NNE Induces Greater NIHL Susceptibility

To elucidate the circadian influence on NIHL susceptibility, we established mouse models of DNE and NNE. Mice were exposed to traumatic noise (2–20 kHz at 107 dB SPL for 2 h) at ZT 2–4 (DNE group) and ZT 14–16 (NNE group). ABR thresholds were elevated in both groups after 14 days of noise exposure, with the NNE group exhibiting significantly greater threshold shifts at 16 and 32 kHz *versus* the DNE group (Fig. S1A). Similarly, the decrement of wave I amplitudes at these frequencies was more pronounced in the NNE group (Fig. S1B, C). Notably, no significant difference in ABR thresholds was recorded 1-day post-exposure, suggesting comparable mechanical damage across groups (Fig. S1D). DPOAE thresholds showed analogous trends, with significantly worse outcomes in the NNE group (Fig. S1E).

Structural analysis of cochlear tissues revealed that outer hair cells (OHCs) and stereocilia bundles displayed greater disarray and degeneration in the NNE group, as shown by SEM (Fig. S1F, G). While OHCs in the apex and middle regions appeared normal with minimal loss in the basal turn, the extent of OHC loss did not differ significantly between the DNE and NNE groups (Fig. S1H, I). These results confirmed that NNE exacerbates cochlear functional impairment and structural damage, inducing greater NIHL susceptibility.

### Single-Cell RNA Sequencing Highlights Macrophage Enrichment in the Cochlear Immune Response to Noise

**Fig. 1:**
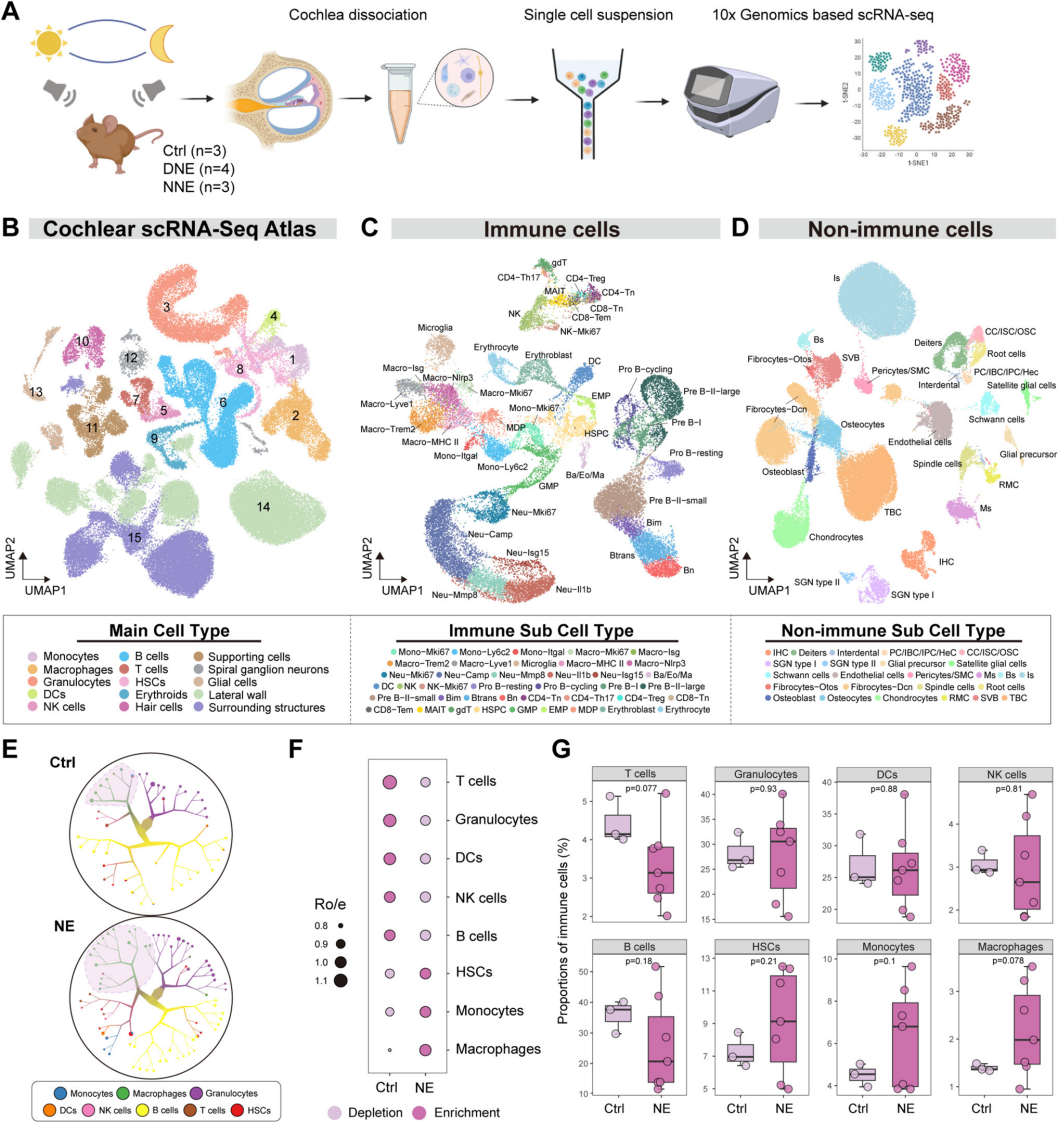
Single-cell atlas of immune and non-immune cells from the cochleae of control and noise-exposed mice. **A** Flowchart of the scRNA-seq experimental design of this study (created using Biorender). **B** UMAP plot displaying the overview of 15 cell clusters including immune and non-immune cells derived from the cochleae of control and noise-exposed mice. The clusters are labeled based on the identified cell subsets, determined by their specific gene expression patterns (see also Fig. S2A and Table S1). **C** UMAP plot showing the clustering of immune cells within cochleae before and after noise exposure. **D** UMAP plot showing the clustering of non-immune cells within cochleae before and after noise exposure. **E** Immune cell tree structure, organized by cell subsets in cochlear samples from control (Ctrl) and noise-exposed (NE) groups (generated using TooManyCells). **F** Ro/e (ratio of observed to expected cell number) analysis of immune cells in the Ctrl and NE groups using the STARTRAC-dist algorithm. **G** Proportions of immune cell populations in the Ctrl and NE groups.

To investigate the cellular and molecular mechanisms underlying the circadian modulation of NIHL, we applied scRNA-seq to mouse cochlear tissues collected before and 3 days after noise exposure (Fig. 1A). Using a droplet-based 10× Genomics platform, we analyzed 97,043 single-cell transcriptomes from pooled cochlear samples (11–12 mice per sample). UMAP analysis identified 15 distinct cell populations, comprising 9 immune and 6 non-immune clusters (Figs 1B; S2A; Table S1). Immune cells accounted for ~40% of the total population, with non-immune cells accounting for ~60% (Fig. S2B, C).

The cochlear single-cell atlas comprised both immune and non-immune clusters, with immune cells including monocytes, macrophages, granulocytes, dendritic cells, natural killer cells, B cells, T cells, hematopoietic stem cells, and erythroid cells (Fig. 1C), while non-immune cells encompassed HCs, supporting cells, spiral ganglion neurons (SGNs), glial cells, and cells from the lateral wall and surrounding structures (Fig. 1D). Cluster identity was determined based on the expression of established lineage-specific marker genes (Fig. S2A; Table S1). The extensive clustering highlighted the cellular heterogeneity of the cochlea, underscoring its dynamic role in the auditory system.

To evaluate noise-induced changes in the immune population, we applied the TooManyCells algorithm [32] for clustering analysis and Ro/e analysis [33] to track changes in cell type proportions under different conditions. The Ro/e normalization adjusted for technical variations, allowing robust comparisons. Macrophages and monocytes exhibited significant enrichment post-exposure, emerging as the primary immune responders (Fig. 1E, F). Among non-immune populations, SGNs showed the most notable transcriptional alterations, underscoring their involvement in cochlear injury (Fig. S2D). Despite these changes, the proportions of non-immune cell populations remained stable (Fig. S2E, F), emphasizing the unique susceptibility of immune cells to noise exposure.

### NNE Amplifies Macrophage Recruitment and Activation

**Fig. 2:**
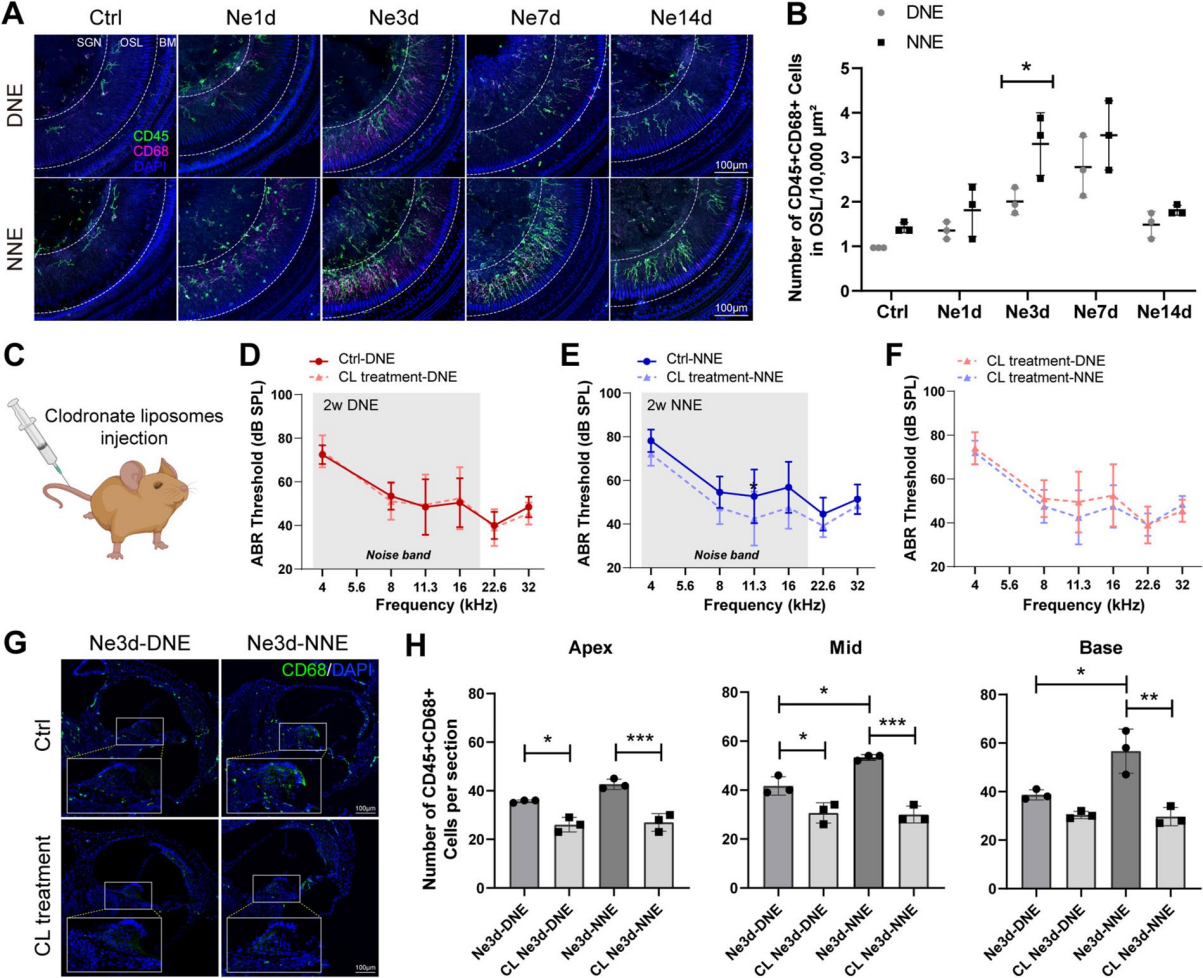
Macrophage recruitment and activation in response to noise exposure, particularly after NNE. **A** Representative confocal images of activated OSL macrophages in the middle turn of cochleae at pre-exposure days 1, 3, 7, and 14 (Ctrl, Ne1d, Ne3d, Ne7d, and Ne14d) after DNE and NNE. Macrophages are stained with CD45 (green) and CD68 (pink). Scale bars, 100 μm. For representative images of other OSL and BM macrophages, see also Fig. S3A–J. **B** Quantification of CD45^+^CD68^+^ macrophages in the OSL of the middle turn. Macrophage counts increase significantly after 3 days of exposure, particularly in the NNE group. Data are presented as the mean ± SD from 3 mice per group, replicated across three independent noise exposures (ns, no significant difference, *P < 0.05, two-way ANOVA, Bonferroni *post hoc* test). **C** Schematic of macrophage depletion using CLs *via* caudal vein injection. **D–F** ABR thresholds were measured 14 days after DNE and NNE, comparing CL treatment with control. Grey represents the range of noise frequencies. Data are presented as the mean ± SD, replicated across 3–4 independent noise exposures per group (*P < 0.05, two-way ANOVA, Bonferroni *post hoc* test, *n* = 10 for Ctrl-DNE, *n* = 10 for CL treatment-DNE, *n* = 11 for Ctrl-NNE, and *n* = 12 for CL treatment-NNE). **G** Representative confocal images of macrophages stained by CD68 (green) in the mid-modiolar axis at 3 days after DNE and NNE, comparing CL treatment with control. Scale bars, 100 μm. **H** Numbers of macrophages per section in the apical, middle, and basal turns. Data are presented as the mean ± SD from 3 mice per group, replicated across three independent noise exposures (*P < 0.05, **P < 0.01, ***P < 0.001, one-way ANOVA, Bonferroni *post hoc* test).

Noise exposure induced the immediate and persistent recruitment of cochlear macrophages, lasting up to 14 days post-exposure in both DNE and NNE groups (Figs 2A, B; S3A–J). Notably, macrophage recruitment and activation were significantly more pronounced in the NNE group. Quantitative analysis revealed increased numbers of CD45^+^CD68^+^ macrophages in the OSL and BM regions, with the greatest differences at 3 days post-exposure (Figs 2A, B; S3A–J).

The enhanced macrophage response in the NNE group was consistent across cochlear regions, particularly in the middle and basal turns (Figs 2A, B; S3C, D, G–J). These results identified 3 days post-exposure as a critical time point for differentiating the immune responses to DNE and NNE. This aligns with previous studies reporting that macrophage activation peaks within 3–7 days post-exposure [18, 44]. Collectively, these findings underscore the heightened inflammatory response to NNE, contributing to greater NIHL susceptibility.

### Macrophages are Essential Mediators of Circadian Susceptibility to NIHL

Immunofluorescence staining revealed a significant increase in cochlear macrophages following NNE. To investigate the role of macrophages in NIHL, we conducted macrophage depletion experiments using CLs, which are selectively phagocytosed by macrophages, inducing apoptosis and effectively reducing their population [45, 46]. CLs have also been used in cochlear studies to investigate macrophage functions [47, 48]. After macrophage depletion, hearing thresholds in the NNE group returned to daytime levels by 14 days post-exposure, whereas the DNE group exhibited no significant recovery (Fig. 2C–F). Immunofluorescence confirmed reduced macrophage numbers in both groups at 3 days post-exposure, with the difference in CD45^+^CD68^+^ cell counts between the two groups significantly diminished after depletion (Fig. 2G, H).

These findings suggest that the differences in NIHL susceptibility between DNE and NNE are primarily mediated by macrophage activity. The absence of significant hearing recovery in the DNE group could reflect the dual protective and damaging roles of macrophages in the cochlea [19, 47]. This dual role is context-dependent, as macrophages may contribute to tissue repair and modulate inflammation under certain conditions [19, 49, 50]. In the DNE group, macrophage-mediated repair processes might have counteracted inflammation-induced damage, leading to a relatively stable cochlear environment. Conversely, macrophages in the NNE group may have exhibited a more pro-inflammatory phenotype, exacerbating cochlear injury.

### Circadian Influence on Macrophage-Mediated Inflammatory Responses in NIHL

**Fig. 3:**
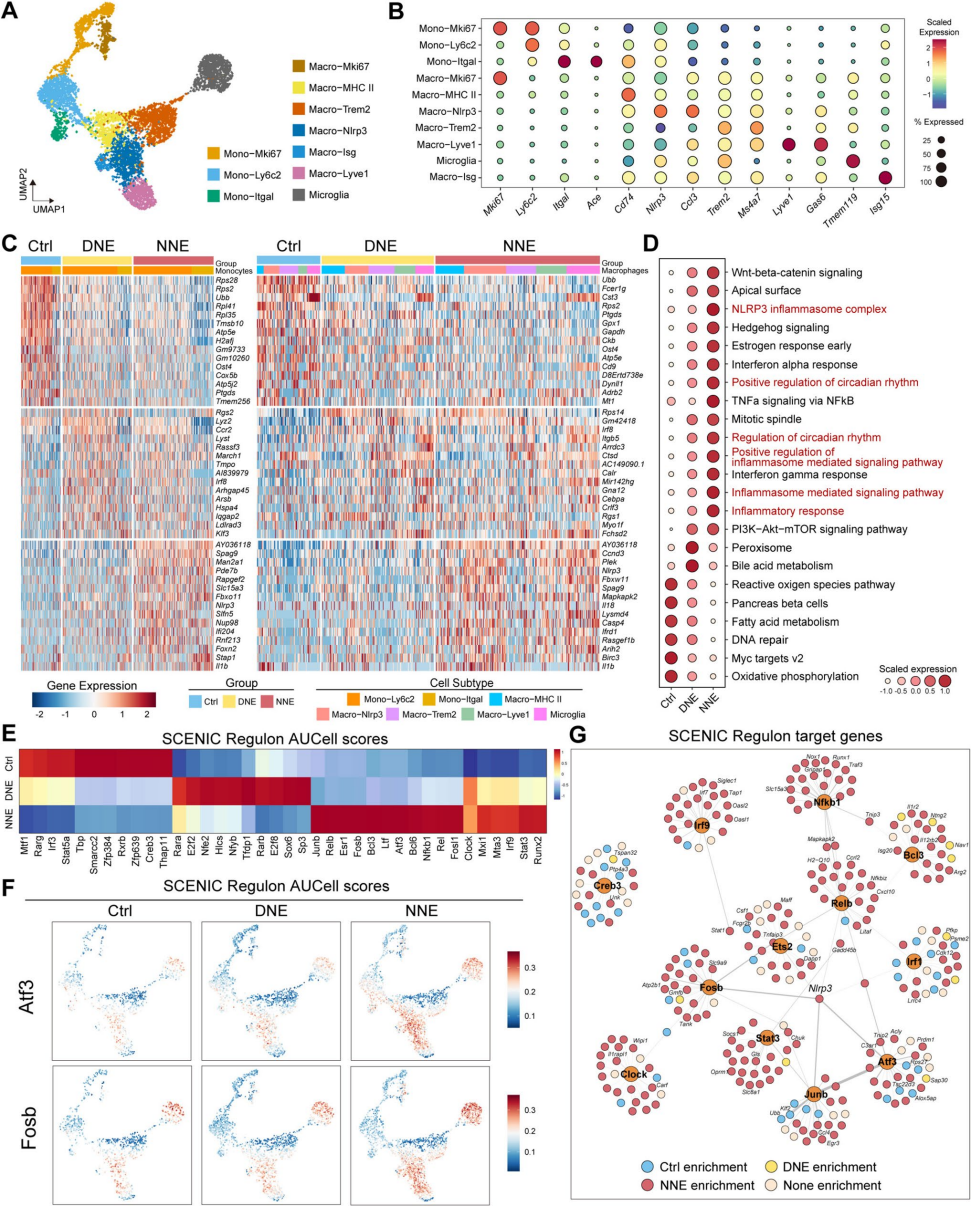
Mononuclear phagocytes and their regulatory networks in response to noise exposure, particularly in NNE. **A** UMAP plot displaying mononuclear phagocyte subsets, including three monocyte subsets and seven macrophage subsets, color-coded according to cell subsets. **B** Dot plot showing cell cluster-specific marker genes in monocyte and macrophage subsets. Color indicates the scaled average expression and dot size represents the proportion of cells expressing these genes. **C** Heatmap displaying the scaled expression values of the top 15 DEGs in subsets of monocytes and macrophages across control, DNE, and NNE groups. **D** Dot plot showing the top enriched pathways across the three groups. **E** Heatmap showing the regulon AUCell scores for mononuclear phagocytes across the three groups, calculated using the SCENIC algorithm. **F** UMAP plot showing the regulon activity of *Atf3* and *Fosb* in mononuclear phagocyte subsets. **G** Cytoscape graph illustrating the regulatory networks composed of regulons and their target genes involved in mononuclear phagocyte development and function. For visualization purposes, only 25 randomly-selected target genes for each region are shown, with each target color-coded by group specificity. See also Fig. S4B and Table S2.

Our data demonstrated that macrophages not only mediate the cochlear immune response but also play a crucial role in circadian variations in the severity of hearing loss. To further characterize macrophage responses, we applied dimensionality reduction and re-clustering analysis of mononuclear phagocytes (monocytes and macrophages) at 3 days post-exposure. This analysis identified 10 distinct populations of mononuclear phagocytes, categorized into three monocyte subtypes and seven macrophage subtypes based on DEGs (Fig. 3A, B; Table S1). Our analysis revealed a high level of diversity among mononuclear phagocytes within the cochlea, including *Mki67*^+^, *Ly6c2*^+^, and *Itgal*^+^ monocytes, as well as *Mki67*^+^, major histocompatibility complex (MHC) II^+^, *Nlrp3*^+^, *Trem2*^+^, *Lyve1*^+^, and *Isg*^+^ macrophages and microglia.

Notably, the MHC II^+^ subset expressed genes associated with antigen presentation, such as *Cd74*, *H2-Aa*, and *H2-Eb1*, while *Lyve1*^+^ macrophages expressed the M2-like markers *Cd163* and *Mrc1* (Figs 3B; S4A, B). Microglial clusters, the specialized macrophages of the central nervous system [51], were characterized by unique markers including *Tmem119*, *Sparc,* and *Siglech* (Figs 3B; S4A, B). Proliferative populations (*Mki67*^+^) were found among both monocytes and macrophages, indicating active cell division and differentiation at 3 days post-exposure (Figs 3A; S4A).

Heatmap clustering of DEGs highlighted distinct transcriptional profiles among macrophage subclusters. *Nlrp3*^+^ macrophages showed upregulation of genes associated with inflammatory pathways and chemokines, indicating a prominent role in immune and inflammatory responses following NNE. Conversely, *Trem2*^+^ macrophages displayed lower expression of these genes, suggesting opposing functions in inflammation (Figs 3C; S4B).

In addition, both daytime and nighttime mononuclear phagocytes exhibited overlap in the upregulated genes associated with inflammation and apoptosis (Fig. 3C), suggesting a common activation pattern regardless of the timing of noise exposure. This shared gene expression profile underscores the robust and consistent activation of cochlear innate immune cells in response to acoustic stress, while the distinct roles of *Nlrp3*^+^ and *Trem2*^+^ macrophages highlight the complexity of the immune response in the cochlea.

### Circadian Regulation of the NLRP3 Inflammasome Pathway in Cochlear Macrophages

Pathway analysis revealed significant upregulation of the NLRP3 inflammasome and circadian rhythm pathways in macrophages following noise exposure, with more pronounced activation in the NNE group (Fig. 3D). These findings suggest that the timing of noise exposure modulates macrophage-mediated inflammatory responses, contributing to differential NIHL susceptibility between daytime and nighttime.

To further explore the regulatory mechanisms underlying these responses, we analyzed upstream transcription factors (TFs) using SCENIC [36], a tool for single-cell regulatory network inference and clustering. In the NNE group, TFs associated with the NLRP3 inflammasome, such as *Fosb*, *Junb*, *Atf3*, and *Relb*, were prominently upregulated (Fig. 3E). In contrast, TFs associated with cell-cycle regulation, including *Rara*, *Rarb*, *E2f2*, and *Nfyb*, were more prevalent in the DNE group (Fig. 3E). *Atf3*, a well-documented regulator of noise-induced SGN damage [13], emerged as the driver in the NNE group, orchestrating a network of genes linked to inflammation and cellular stress responses (Fig. 3F, G).

Interestingly, TFs such as *Irf9* and *Stat3*, although upregulated in the NNE group, were primarily involved in interferon signaling and did not directly influence the NLRP3 pathway (Fig. 3G). These findings underscored the complex regulatory networks influencing macrophage behavior in response to noise exposure. Collectively, these findings emphasize the interplay between circadian factors and macrophage-driven inflammation in shaping the cochlear immune response and its contribution to NIHL.

### NNE Amplifies NLRP3 Inflammasome Activation in Cochlear Macrophages

Building on the distinct transcriptional landscapes between the DNE and NNE groups, we focused on the NLRP3 inflammasome complex. This pathway, a central component of the innate immune response, mediates inflammatory responses by promoting the release of pro-inflammatory cytokines such as IL-1β and IL-18 in response to cellular stress [52]. While extensively studied in other inflammatory and neurodegenerative conditions [53, 54], the circadian modulation of this pathway in cochlear macrophages remains largely unexplored.

**Fig. 4:**
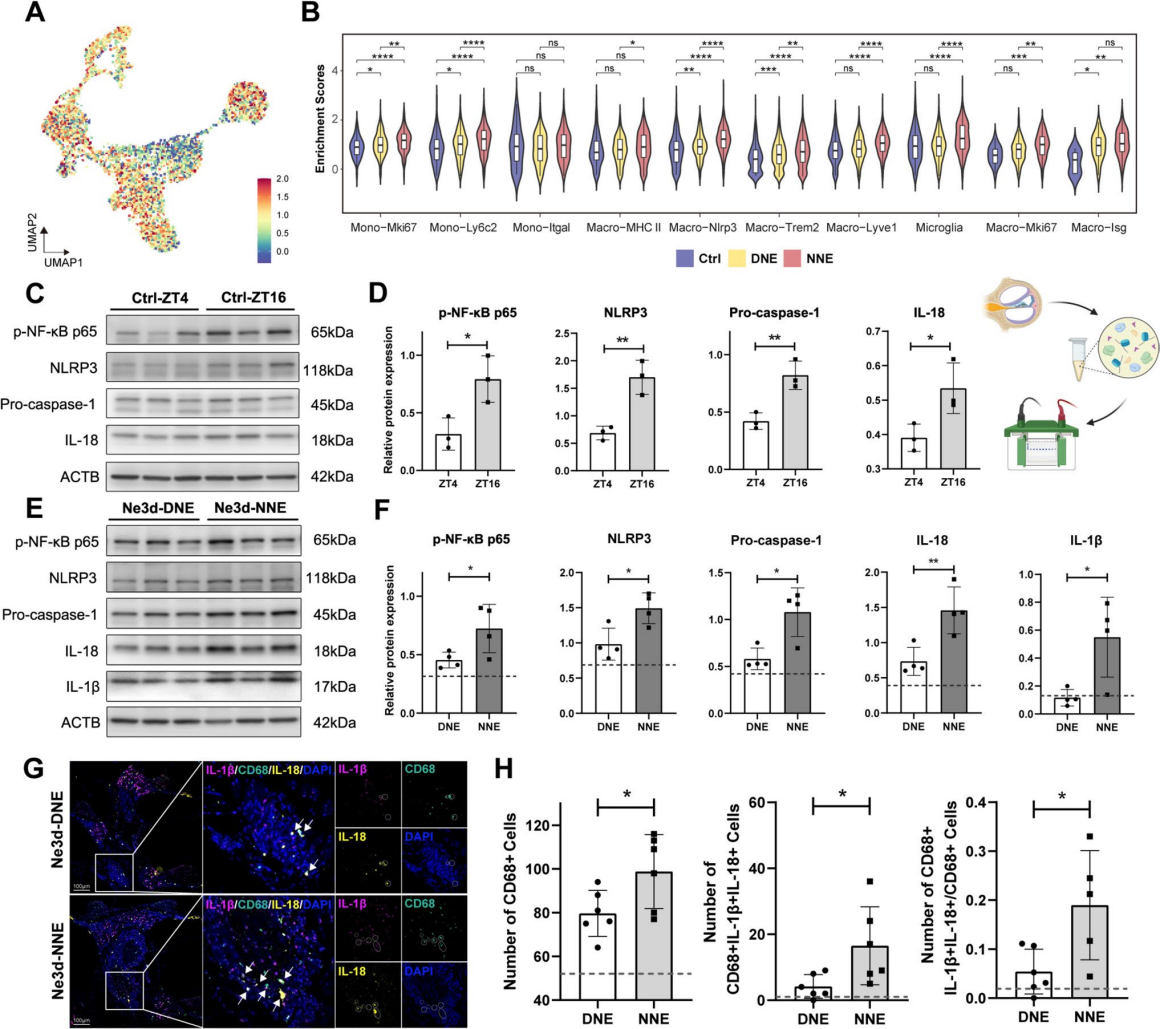
Activation of the NLRP3 inflammasome pathway in mononuclear phagocytes in response to noise exposure, particularly in NNE. **A** UMAP plot showing the signaling pathway of the NLRP3 inflammasome complex in mononuclear phagocytes subsets. **B** Violin plots displaying the enrichment score of the NLRP3 inflammasome complex signaling pathway in subsets of monocytes and macrophages across the three groups. **C, D** Representative western blots and statistical analysis of p-NF-κB p65, NLRP3, pro-caspase-1, and IL-18 protein expression levels at ZT4 and ZT16. Protein levels are normalized to ACTB (β-Actin). Unpaired *t*-test. Data are presented as the mean ± SD from 3 mice per group, replicated across three independent noise exposures. **E, F** Representative western blots and statistical analysis of p-NF-κB p65, NLRP3, pro-caspase-1, IL-1β, and IL-18 protein expression levels at 3 days after DNE and NNE. Protein levels are normalized to ACTB. The dashed line represents the protein expression level of the corresponding protein at ZT4. Unpaired *t-*test. Data are presented as the mean ± SD from 4 mice per group, replicated across four independent noise exposures. **G** Representative images of mIHC staining for IL-1β (pink), CD68 (green), IL-18 (yellow), and DAPI (blue), used to quantify the spatial co-localization of NLRP3 signaling pathway molecules and macrophages in the cochlea at 3 days after DNE and NNE. Arrows indicate CD68^+^IL-1β^+^IL-18^+^ cells. Scale bars, 100 μm. For representative images of the control group, see Fig. S4E. **H** Quantification of CD68^+^ cells, CD68^+^IL-1β^+^IL-18^+^ cells, and the proportion of CD68^+^IL-1β^+^IL-18^+^ cells among total CD68^+^ cells in the DNE and NNE groups. The dashed line represents the corresponding quantification in the control group. Data are presented as the mean ± SD from 5–6 mice per group, replicated across three independent noise exposures (*P < 0.05, **P < 0.01, ***P < 0.001, ****P < 0.0001, unpaired *t*-test).

Our findings revealed that the NLRP3 inflammasome signaling pathway was expressed across nearly all mononuclear phagocyte subsets, with robust activation in the NNE group compared to the DNE group (Figs 4A, B; S4C). *Nlrp3* expression was prominently localized to mononuclear phagocytes, with minimal expression in other immune and non-immune cell types (Fig. S4D). Among these subsets, the *Nlrp3*^+^ macrophages exhibited the highest NLRP3 pathway activity, which was markedly upregulated during NNE (Fig. 4B).

To further investigate the functional implications of these findings, we assessed the protein levels of key inflammasome components in the cochlea**.** The baseline expression of p-NF-κB p65, NLRP3, pro-caspase-1, and IL-18 was inherently higher at ZT16 than at ZT4, suggesting a heightened immune state during night-time (Fig. 4C, D). Following noise exposure, these proteins, as well as the inflammatory cytokine IL-1β, were significantly elevated in both the DNE and NNE groups, with the NNE group showing the greatest increases (Fig. 4E, F).

To validate our results at the cellular level, we applied mIHC to quantify the spatial co-localization of NLRP3 signaling molecules within macrophages. Our analysis confirmed a marked increase in CD68^+^IL-1β^+^IL-18^+^ macrophages in the NNE group compared to the DNE group (Figs 4G, H; S4E). These data collectively demonstrate that NNE exacerbates macrophage-driven inflammatory responses, contributing to the heightened susceptibility to NIHL during NNE.

### Targeting the NLRP3-Dependent Inflammation Pathway with CY-09 Attenuates Nocturnal NIHL

**Fig. 5:**
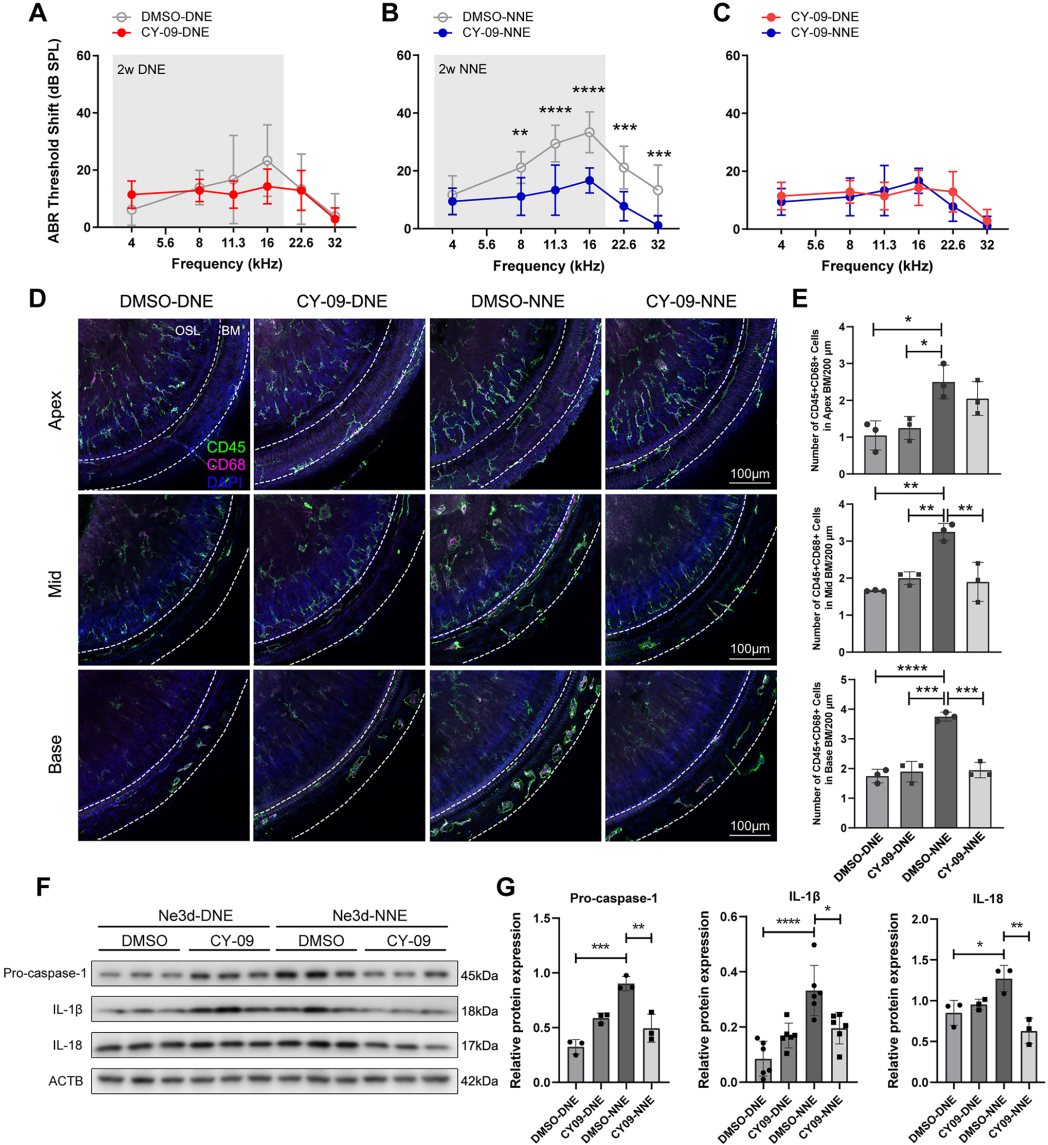
Therapeutic potential of NLRP3 inhibition in the progression of nocturnal NIHL. **A–C** ABR thresholds were measured 14 days after DNE and NNE, comparing the CY-09-treated group and the control group. Grey shading indicates the range of noise frequencies. Data are presented as the mean ± SD, replicated across four independent noise exposures per group (*n* = 9 for DMSO-DNE, *n* = 7 for CY-09-DNE, *n* = 9 for DMSO-NNE, and *n* = 9 for CY-09-NNE; **P < 0.01, ***P < 0.001, ****P < 0.0001, two-way ANOVA, Bonferroni *post hoc* test). **D** Representative confocal images of CD45 (green) and CD68 (pink)-stained macrophages in the BM at 3 days after DNE and NNE. Scale bars, 100 μm. **E** Numbers of CD45^+^CD68^+^ cells in the BM at 3 days after DNE and NNE. Data are presented as the mean ± SD from 3 mice per group, replicated across three independent noise exposures (*P < 0.05, **P < 0.01, ***P < 0.001, ****P < 0.0001, one-way ANOVA, Bonferroni *post hoc* test). **F, G** Representative western blots and statistical analysis of pro-caspase-1, IL-1β, and IL-18 protein expression at 3 days after DNE and NNE. Protein levels are normalized to ACTB. Data are presented as the mean ± SD from 3–6 mice per group, replicated across three independent noise exposures (*P < 0.05, **P < 0.01, ***P < 0.001, ****P < 0.0001, one-way ANOVA, Bonferroni *post hoc* test).

Considering the pivotal role of the NLRP3 inflammasome in circadian NIHL susceptibility, we evaluated the therapeutic potential of CY-09, a selective NLRP3 inhibitor. CY-09 directly binds to the ATP-binding motif of the NLRP3 NACHT domain, inhibiting ATPase activity and inflammasome activation [43]. It has shown efficacy in mitigating inflammation in disease models of conditions such as Alzheimer’s disease and arthritis [55, 56]. In our study, CY-09 administration significantly attenuated NIHL in the NNE group, restoring ABR thresholds to levels comparable to those in the DNE group (Fig. 5A–C). By contrast, only minor recovery in hearing loss was noted in the DNE group. These findings suggest that CY-09 effectively mitigates the heightened auditory damage associated with NNE.

To further investigate the mechanisms underlying these effects, we assessed cochlear macrophage activation and infiltration. A notable reduction in CD45^+^CD68^+^ macrophages in the BM regions was found in the NNE group following CY-09 administration (Fig. 5D, E). The levels of NLRP3 inflammasome components and associated cytokines, including IL-1β and IL-18, were significantly lower in the NNE group after treatment, along with decreased pro-caspase-1 expression (Fig. 5F, G). This reduction is likely due to CY-09’s inhibition of NLRP3 inflammasome activation, which decreases the secretion of pro-inflammatory cytokines, particularly IL-1β and IL-18, essential for macrophage recruitment and activation.

These results indicate that CY-09 effectively suppresses NLRP3 inflammasome activation, dampens macrophage-driven inflammatory responses, and reduces cochlear inflammation and damage, particularly following nocturnal noise exposure. By preserving cochlear function and mitigating circadian-sensitive inflammatory damage, CY-09 highlights the therapeutic potential of targeting the NLRP3 inflammasome in NIHL.

### Macrophage-Driven Immune Interactions and SGN Type I Vulnerability Following NNE

To understand how macrophage-mediated immune responses influence cochlear damage, we examined interactions between immune and non-immune cells. Non-immune cell proportions remained stable (Fig. S2E), except for a slight reduction in lateral wall cells in the NNE group (Fig. S5A), possibly due to immediate disruptions in cochlear blood flow and endolymphatic homeostasis after noise exposure [57].

**Fig. 6:**
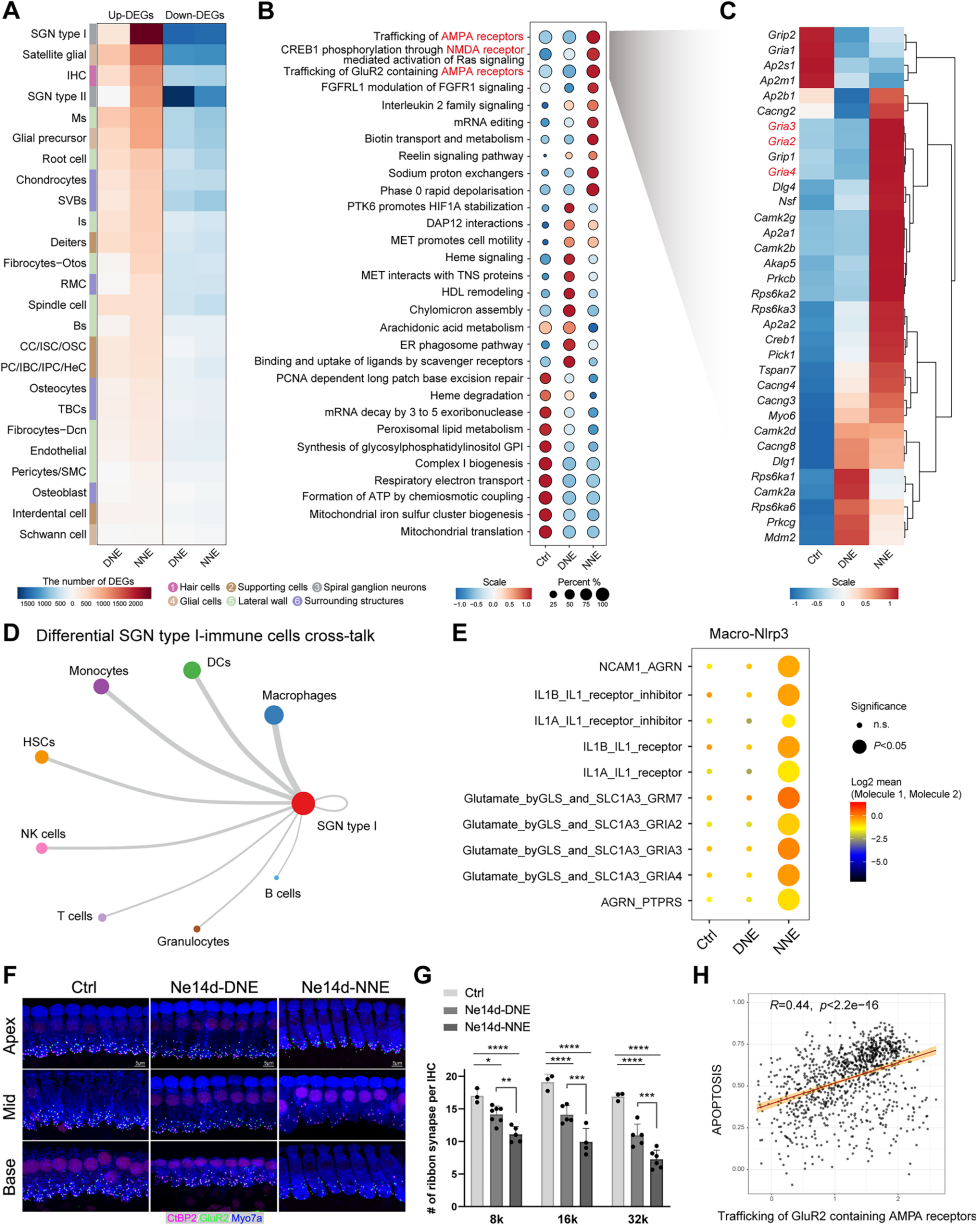
Cell-cell interactions between SGN type I and immune cells. **A** DEG analysis shows upregulated and downregulated genes in non-immune cell subsets in the DNE and NNE groups (see also Table S3). **B** Dot plot showing pathway enrichment analysis of SGN type I, highlighting significant involvement in AMPA and NMDA receptor pathways. **C** Heatmap displaying DEGs involved in AMPA and NMDA receptor pathways across control, DNE, and NNE groups. **D** Ranked differential cross-talk analysis of SGN type I and immune cells, with macrophages ranking first among all ligand-receptor pairs, calculated using CellPhoneDB. **E** NNE upregulates ligand-receptor cross-talk between *Nlrp3*^+^ macrophages and SGN type I within cochlear cells (y-axis, ligand and receptor name; x-axis, the tissue; circle size, the log-normalized *P* value; color intensity, the log-transformed mean expression of ligand and receptor). **F** Representative confocal images of IHCs co-labeled with the presynaptic marker CtBP2 (pink) and the postsynaptic marker GluR2 (green) before exposure, and 14 days after DNE and NNE. Scale bars, 5 μm. **G** Quantitation of ribbon synapses per IHC. Puncta co-labeled with CtBP2 and GluR2 are counted as ribbon synapses. The average number of synapses per IHC is calculated from 1–2 fields of view per frequency per mouse. Data are presented as the mean ± SD, replicated across three independent noise exposures per group. *n* = 3 for the Ctrl group, *n* = 5–7 for the Ne14d-DNE group, *n* = 4–6 for the Ne14d-NNE group. *P < 0.05, **P < 0.01, ***P < 0.001, ****P < 0.0001, two-way ANOVA, Bonferroni *post hoc* test,. **H** Correlation coefficients between the score for trafficking GluR2-containing AMPA receptors and the apoptosis score in SGN type I.

Among non-immune cell populations, SGN type I displayed the most pronounced transcriptional changes and was one of the most affected cell types in the NNE group (Figs 6A; S5B). GO analysis further revealed increased synaptic activity, alongside the downregulation of pre- and postsynaptic translation pathways in the NNE group (Fig. S5C). Pathway analysis identified the upregulation of glutamate excitotoxicity pathways, including AMPA receptor trafficking, CREB1 phosphorylation *via* the activation of NMDA receptor-triggered Ras signaling, and GluR2-containing AMPA receptor trafficking (Fig. 6B; Table S3). Phase 0–2 signaling pathways essential for SGN action potential generation were also elevated in the NNE group (Table S4). These results suggest that nocturnal acoustic trauma may lead to elevated cochlear sensitivity or excitotoxicity, resulting in more severe synaptic damage and hearing loss.

Upregulated DEGs within the glutamate receptor pathways, including *Gria2–4*, were significantly upregulated in the NNE group (Fig. 6C). Interestingly, *Gria2–4* expression levels were comparable in the DNE and NNE groups prior to noise exposure but showed a slight increase in both groups 1–day post-exposure, as revealed by bulk RNA-seq (Fig. S6A). These findings suggest that the excitotoxicity in the NNE group was amplified by the inflammatory response to noise exposure.

Ligand–receptor cross-talk analysis revealed that SGN type I preferentially interacted with macrophages, particularly the *Nlrp3*^+^ subtype, *via* IL-1β receptor-ligand interactions (Figs 6D, E; S6B). This interaction may contribute to synaptopathy, as evidenced by the significant loss of ribbon synapses in the NNE group (Fig 6F, G). Ribbon synapses, critical for auditory signal transmission from IHCs to SGNs, were severely disrupted, reflecting the enhanced susceptibility of SGN type I to nocturnal noise.

Moreover, macrophages also ranked highest in ligand-receptor interactions with IHCs (Fig. S6C), suggesting strong interactions between these cell types during acoustic insult. Glutamate excitotoxicity was positively correlated with SGN type I apoptosis (Fig. 6H), exacerbating the hearing loss. Treatment with CY-09 significantly improved synaptic integrity in the NNE group, reducing synaptopathy and preserving auditory function (Fig. S6D, E). These findings highlight the critical role of macrophage-driven inflammation in cochlear damage and underscore the therapeutic potential of NLRP3 inhibition in mitigating synaptic and neural deficits.

## Discussion

Circadian rhythm significantly influences susceptibility to NIHL; however, the underlying mechanisms of this temporal variability remain poorly understood. Our study sheds light on this phenomenon by identifying macrophages as central mediators of circadian-specific immune response to acoustic trauma. Utilizing scRNA-seq to profile cochlear cells, we identified macrophages as the most responsive immune cell population, with pronounced activation of the NLRP3 inflammasome signaling pathway during NNE. This activation promotes the release of the pro-inflammatory cytokines IL-1β and IL-18, exacerbating glutamate-mediated excitotoxicity and synaptic damage. These findings highlight the role of macrophages in cochlear immune defense and injury, particularly under circadian modulation.

Recent studies have implicated the NLRP3 inflammasome in NIHL, providing foundational insights into cochlear inflammation. For instance, Sai *et al*. demonstrated upregulation of NLRP3 inflammasome signaling following noise exposure, validating its activation at the protein level [58]. However, their study did not identify the cellular origins of NLRP3 activation or explore its role in immune regulation. Similarly, Pan *et al.* investigated macrophage involvement in NIHL, highlighting the TLR4-NLRP3 axis and applying dual fluorescent labeling to differentiate tissue-resident macrophages from bone marrow-derived infiltrating macrophages [44]. While these studies corroborate our findings regarding NLRP3 inflammasome activation and macrophage involvement, they lacked the single-cell resolution to rule out contributions from other immune cell types and did not delineate the broader immune landscape or provide a detailed pathway linking macrophage activation to cochlear injury.

By leveraging scRNA-seq, we uncovered novel insights into the cellular and molecular immune mechanisms driving NIHL. We provided a comprehensive profile of cochlear immune cells at single-cell resolution, revealing detailed immune dynamics under noise exposure. We not only confirmed the pivotal role of NLRP3 inflammasome activation in NIHL but also uncovered its circadian-specific regulation within macrophages. By integrating advanced bioinformatic analyses with experimental validation, we demonstrated that macrophage-driven inflammatory pathways exhibit distinct temporal dynamics, with heightened activation during NNE. Importantly, our findings provide mechanistic insights into how NLRP3 activation amplifies cochlear damage and offer a framework for developing time-sensitive therapeutic interventions targeting macrophage-mediated inflammation.

The limited efficacy of CL and CY-09 in the DNE group can be attributed to several factors. First, the DNE group exhibited relatively mild cochlear damage, potentially due to lower cochlear susceptibility during the daytime. This resulted in less severe inflammation and, consequently, a reduced role for macrophage-mediated injury. Second, the balance between the protective and damaging roles of macrophages may have been skewed towards repair and tissue maintenance during DNE, limiting the effectiveness of interventions targeting macrophages and the NLRP3 inflammasome. In contrast, the NNE group, with more pronounced cochlear damage, likely experienced a more pro-inflammatory macrophage phenotype, making CL and CY-09 treatment more effective in mitigating hearing loss. Lastly, circadian variations in cochlear immune responses may contribute to these differences, with more pronounced inflammatory responses and greater macrophage activation in the NNE group, making CY-09 treatment more effective in that context. These findings underscore the complexity of macrophage functions in cochlear injury and highlight the significance of circadian variations in immune responses when developing therapeutic strategies for NIHL.

Pharmacological inhibition of the NLRP3 inflammasome with CY-09 further validated its central role in NIHL susceptibility. CY-09 effectively mitigated cochlear inflammation, reduced macrophage recruitment, preserved synaptic integrity, and alleviated auditory damage during NNE. These results underscore the therapeutic potential of targeting NLRP3 in NIHL. Notably, the NLRP3 inflammasome has also been implicated in age-related hearing loss, drug-induced ototoxicity, and other immune-related auditory disorders [59–61]. This suggests that NLRP3 inhibitors represent a unifying therapeutic strategy to address a spectrum of hearing loss conditions driven by immune dysregulation.

IL-1β has been reported to play a critical role in enhancing glutamate excitotoxicity across various organ systems, particularly in the central nervous system. In conditions like multiple sclerosis and Alzheimer's disease, IL-1β exacerbates neuronal damage by promoting glutamate release and impairing synaptic stability [62, 63]. Similarly, in the cochlea, our study shows that IL-1β secretion, triggered by NLRP3 inflammasome activation, contributes to excitotoxicity and synaptic loss. These mechanisms, also reported in diseases such as glaucoma [64], highlight the broader role of IL-1β in neuroinflammation and neurodegeneration, suggesting that its role in excitotoxicity extends beyond the cochlea and may be a widespread phenomenon in neuroinflammatory diseases. Beyond macrophages, our study provides a detailed classification of cochlear immune cells, revealing the complex and dynamic nature of the cochlear immune environment. Immune cells in the cochlea not only respond to local stressors, such as noise exposure [65] but also interact with systemic immune pathways [66]. This broader perspective highlights the intricate network of immune regulation within the cochlea and raises questions about how these responses may influence both acute and long-term auditory outcomes. By identifying macrophages as critical mediators, we align our findings with the growing evidence that systemic immune modulation significantly impacts auditory health [67].

While our findings provide valuable insights, they are constrained by certain limitations. First, although nearly 100,000 cells were analyzed, the small sample size (3–4 samples per group) limits the generalizability of our findings. Second, the fragility and vulnerability of OHCs after noise exposure prevented their inclusion in our dataset, as well as in a previous study [22]. Future studies applying advanced techniques such as single-nucleus RNA sequencing [22] and spatial transcriptomics [68] could overcome these challenges, providing a more comprehensive view of cochlear cellular responses, including OHCs [13, 69]. In addition, targeted approaches, such as GFP labeling, could facilitate the isolation and analysis of rare or delicate cell types, refining our understanding of cochlear pathophysiology [70]. Finally, to enhance the clinical relevance of these findings, non-human primate models could serve as a better translational platform due to their closer resemblance to human cochlear anatomy and immune regulation.

In conclusion, our study elucidates the role of macrophage-driven inflammatory pathways in mediating the circadian sensitivity of NIHL. By integrating advanced single-cell technologies with functional validation, we uncover critical mechanisms linking temporal immune regulation to cochlear damage. These findings pave the way for targeted therapeutic strategies to mitigate circadian-specific auditory damage. They also provide a foundation for exploring similar immune mechanisms in other inflammation-related hearing disorders.

## Supplementary Information

Below is the link to the electronic supplementary material.

Supplementary file1 (XLSX 596 kb)

Supplementary file2 (XLSX 2932 kb)

Supplementary file3 (XLSX 3893 kb)

Supplementary file4 (XLSX 91 kb)

Supplementary file5 (PDF 2152 kb)

## Data Availability

The sequencing datasets generated and analyzed during the current study are available in the National Genomics Data Center repository, accessible at https://ngdc.cncb.ac.cn/gsa [71, 72]. Raw single-cell RNA-seq data are available through the National Genomics Data Center with accession number CRA019820, while bulk RNA-seq data have been deposited under accession number CRA019827.
