# Supplementary material for: Single-Cell Sequencing Reveals Circadian Sensitivity of Noise-Induced Hearing Loss Mediated by Macrophage-Driven NLRP3 Inflammasome Activation
Source: Neurosci Bull. 2025 Jul 20;42(2):319–37. doi: 10.1007/s12264-025-01440-1 (PMC12876505; doi:10.1007/s12264-025-01440-1)
Supplement: Supplementary file 5 — Supplementary file5 (PDF 2152 kb) [file 12264_2025_1440_MOESM5_ESM.pdf]

Supplemental Information

Supplemental figure legends

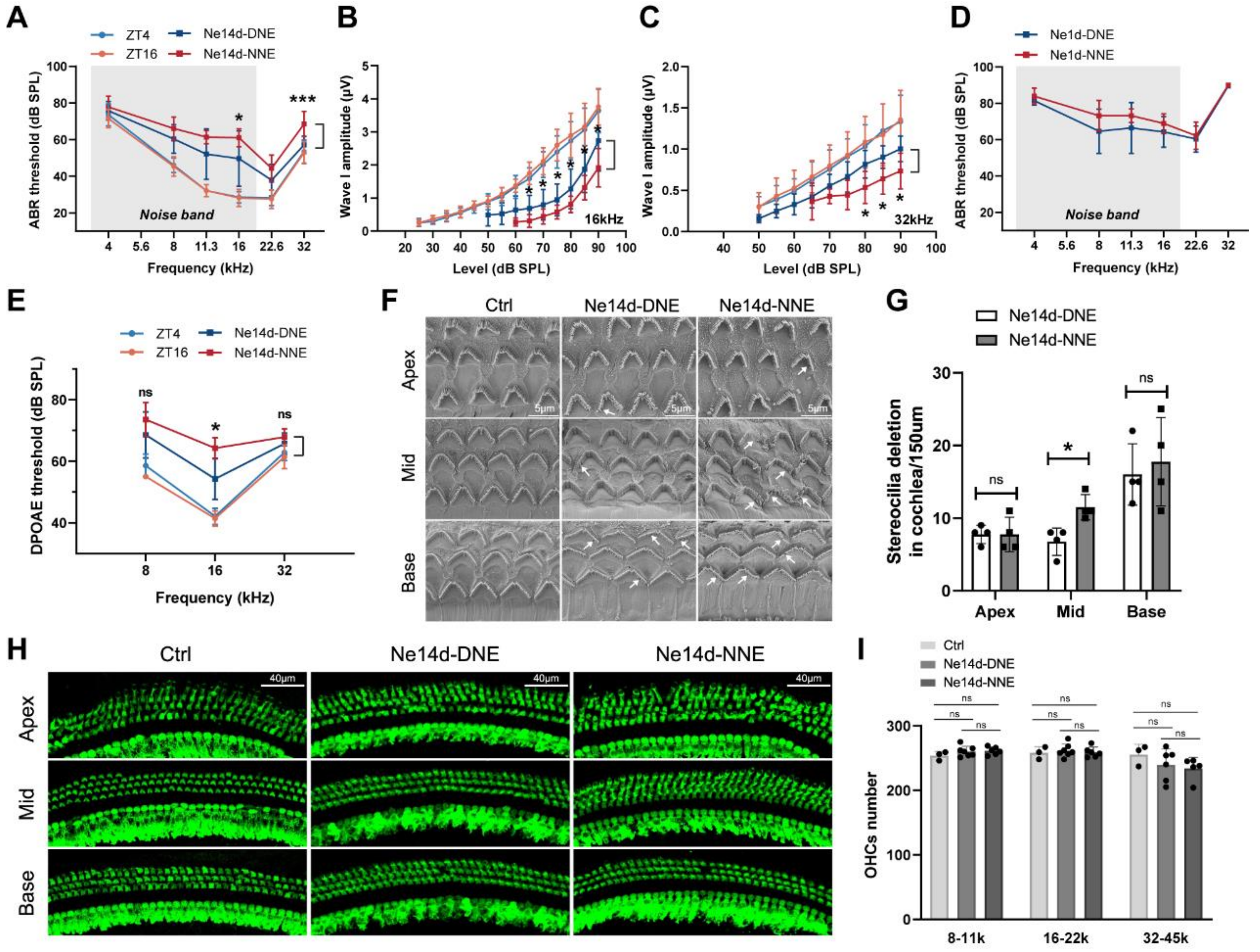

Fig. S1 Auditory function and structure are more vulnerable after nighttime noise exposure. **A** ABR thresholds were measured before and 14 days after DNE and NNE (the grey area represents the range of noise frequencies). **B**, **C** ABR wave I amplitudes, evoked by suprathreshold tones at 16 kHz (**B**) and 32 kHz (**C**). Data are presented as the mean ± SD, replicated across four independent noise exposures per group (*n* = 14 per group; *P <0.05, ***P <0.001, two-way ANOVA, Bonferroni *post hoc* test). **D** ABR threshold measured at 1 day after DNE and NNE. Data are presented as the mean ± SD, replicated across four independent noise exposures per group (*n* = 14 per group). **E** DPOAEs were measured before and 14 days after DNE and NNE. Data are presented as the mean ± SD, replicated across three

independent noise exposures per group (*n* = 7 per group; ns, no significant difference, *P <0.05, two-way ANOVA, Bonferroni *post hoc* test). **F** Representative scanning electron microscopy images of stereocilia before and 14 days after DNE and NNE. Arrows indicate deletion of OHC stereocilia. Scale bars, 5 μm. **G** Quantification of stereocilia deletion after noise exposure, with a more severe loss in the middle turn after NNE. Data are presented as the mean ± SD from 4 mice per group, replicated across four independent noise exposures (ns, no significant difference, *P <0.05, unpaired *t* test). **H** Representative confocal images of OHCs and IHCs immunostained for Myosin VIIa (green). Scale bars, 40 μm. **I** Numbers of OHCs 14 days after DNE and NNE. *n* = 3 for the Ctrl group, *n* = 5–7 for DNE, *n* = 4–6 for NNE. Data are presented as the mean ± SD, replicated across three independent noise exposures per group (ns, no significant difference, two-way ANOVA, Bonferroni *post hoc* test).

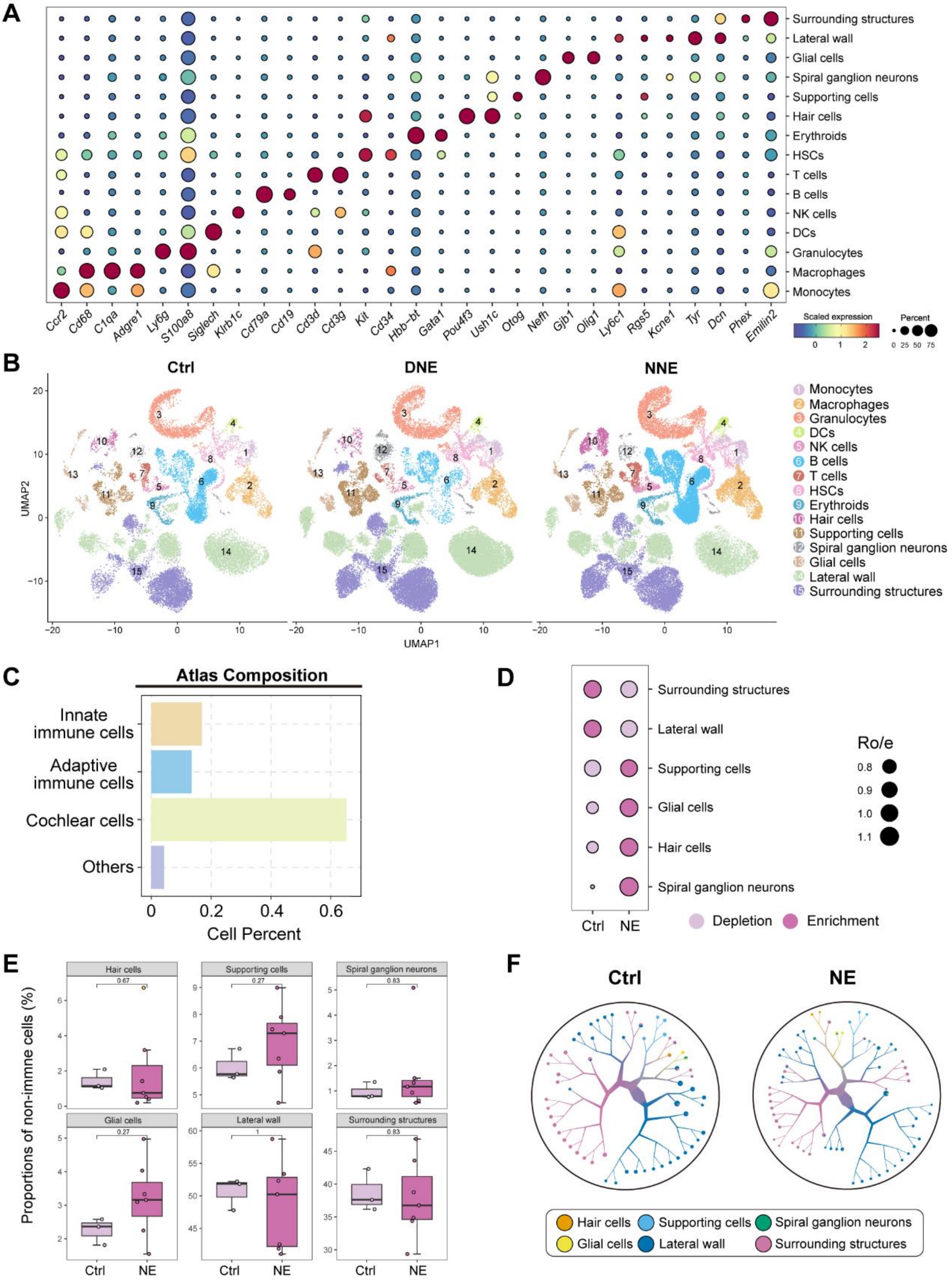

Fig. S2 Single-cell atlas and composition of immune and non-immune cells in cochlear samples from

control, DNE, and NNE groups. **A** Dot plot showing cluster-specific marker genes for 15 main cell types (color indicates scaled average expression and bubble size represents the proportion of cells expressing these genes). **B** UMAP plots demonstrate the absence of batch effects across the control, DNE, and NNE groups. **C** Composition of cochlear scRNA-seq atlas, including innate immune cells, adaptive immune cells, cochlear cells, and other cell types. **D** Ro/e (ratio of observed to expected cell number) analysis of non-immune cells in Ctrl and NE groups using the STARTRAC-dist algorithm. **E** Proportions of non-immune cell populations in Ctrl and NE groups. **F** Non-immune cell tree structure, organized by cell subsets from cochlear samples in Ctrl and NE groups, generated using TooManyCells.

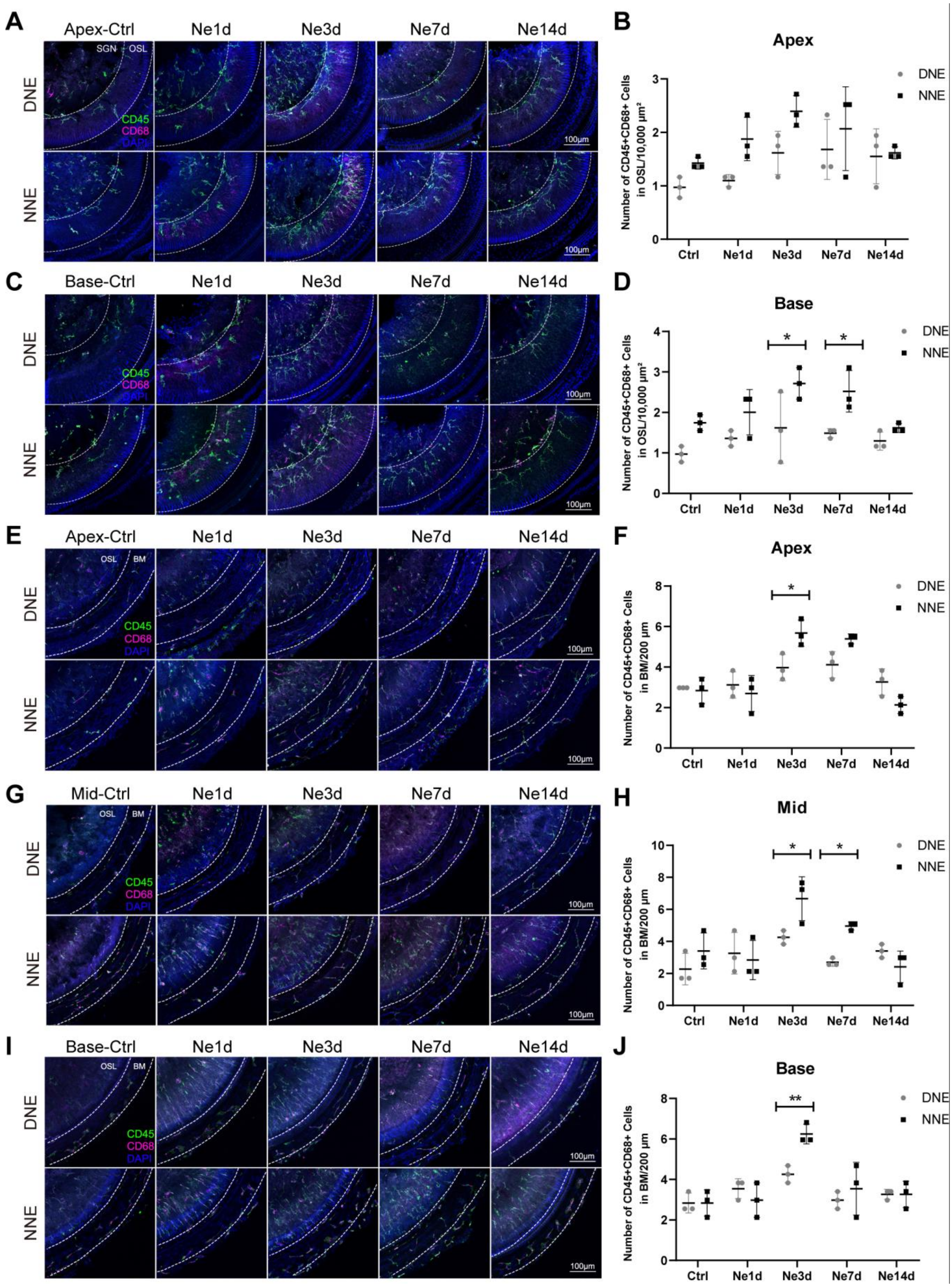
A
Apex-Ctrl
Ne1d
Ne3d
Ne7d
Ne14d
SGN
OSL
DNE
NNE
CD45
CD68
DAPI
100μm
B
Apex
Number of CD45+CD68+ Cells in OSL/10,000 μm²
DNE
NNE
Ctrl
Ne1d
Ne3d
Ne7d
Ne14d
C
Base-Ctrl
D
Base
*
E
Apex-Ctrl
OSL
BM
F
Apex
Number of CD45+CD68+ Cells in BM/200 μm
G
Mid-Ctrl
H
Mid
I
Base-Ctrl
J
Base
**

Fig. S3 Enhanced macrophage recruitment and activation, particularly after NNE. **A–D** Representative confocal images of activated OSL macrophages and statistical analysis of $CD45^+CD68^+$ cell numbers in the apical and basal turns of cochleae before exposure, and 1, 3, 7, and 14 days (Ctrl, Ne1d, Ne3d, Ne7d, Ne14d) after DNE and NNE. Macrophages are stained with CD45 (green) and CD68 (pink). Scale bars, 100 μm. Data are presented as the mean ± SD from 3 mice per group, replicated across three independent noise exposures. *P <0.05, two-way ANOVA, Bonferroni *post hoc* test. **E–J** Representative confocal images of activated BM macrophages and statistical analysis of $CD45^+CD68^+$ cell numbers in the apical, middle, and basal turns of cochleae before exposure, and 1, 3, 7, and 14 days (Ctrl, Ne1d, Ne3d, Ne7d, Ne14d) after DNE and NNE. Macrophages are stained with CD45 (green) and CD68 (pink). Scale bars, 100 μm. Data are presented as the mean ± SD from 3 mice per group, replicated across three independent noise exposures. *P <0.05, **P <0.01, two-way ANOVA, Bonferroni *post hoc* test.

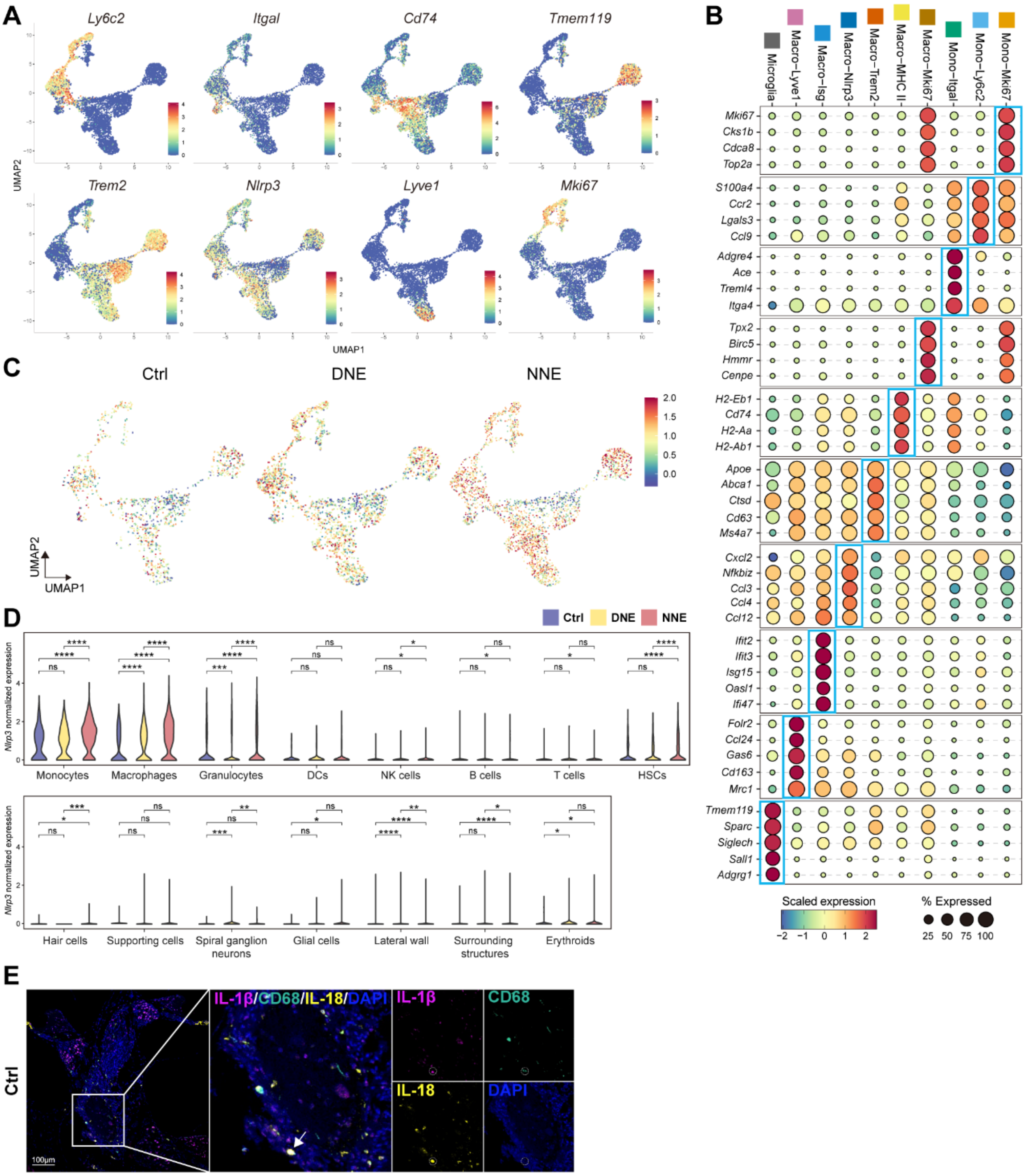

Fig. S4 Mononuclear phagocyte subsets and NLRP3 inflammasome signaling across the control, DNE, and NNE groups. **A** UMAP plots showing the normalized expression of cluster-specific marker genes in mononuclear phagocyte subsets. **B** Dot plot showing cell cluster-specific marker genes in monocyte and macrophage subsets. The dot color indicates scaled average expression and the dot size represents

the proportion of cells expressing these genes. **C** UMAP plots comparing NLRP3 inflammasome activity in mononuclear phagocytes across the three groups. **D** Violin plots showing normalized expression levels of *Nlrp3* in immune and non-immune cell types across the three groups. ns, no significant difference, **P* <0.05, ***P* <0.01, ****P* <0.001, *****P* <0.0001. **E** Representative images of mIHC staining for IL-1β, IL-18, and CD68 in cochlear sections from the Ctrl group. The arrow indicates a $CD68^{+}IL\text{-}1\beta^{+}IL\text{-}18^{+}$ macrophage. Scale bar, 100 μm.

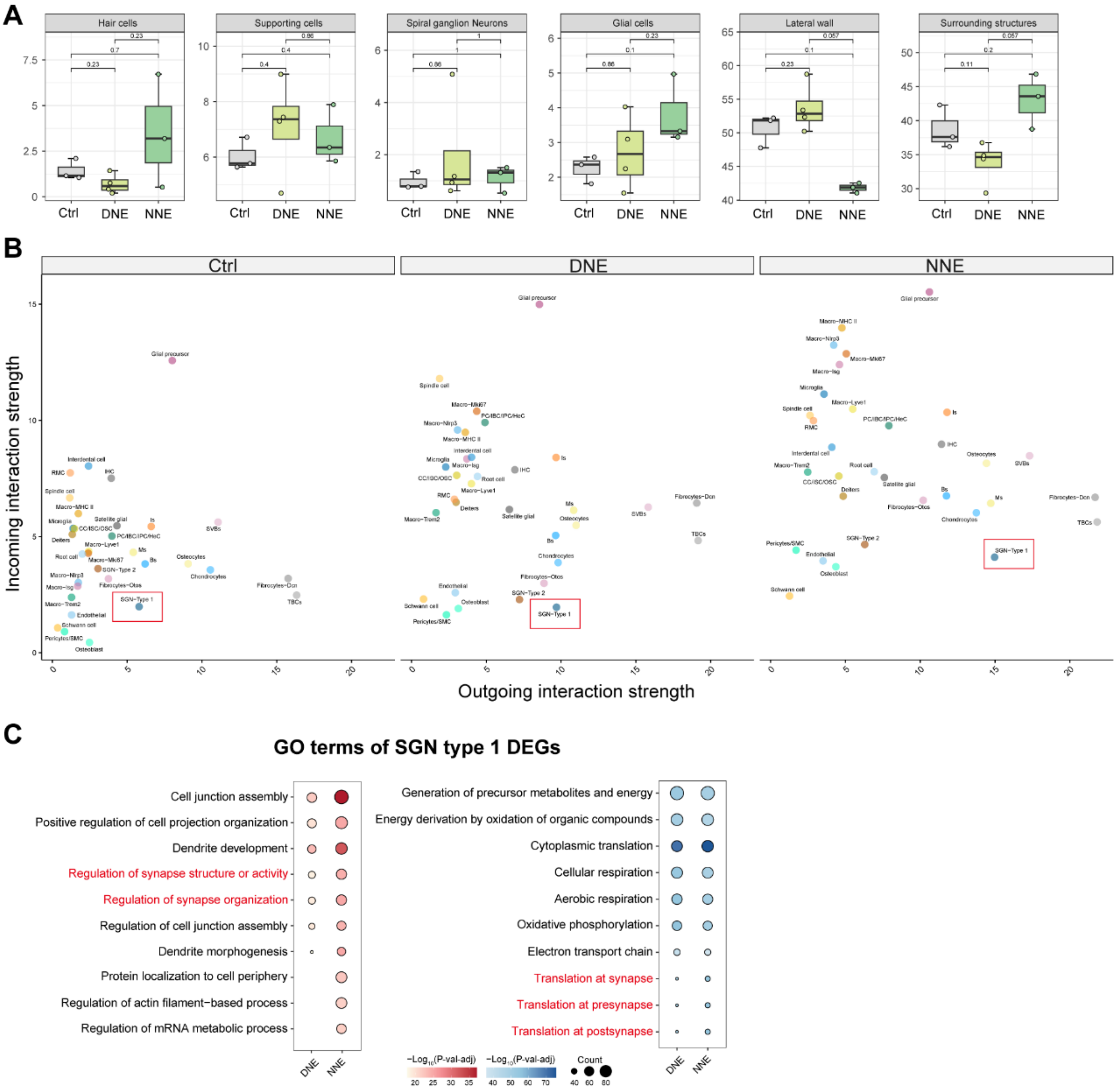

Fig. S5. Non-immune cell populations, cell-cell interactions, and GO enrichment across the control, DNE, and NNE groups. **A** Proportions of non-immune cell populations across the three groups. **B** Cell-cell interaction network plot displaying the interaction between macrophages and non-immune cells across the three groups. **C** Dot plots showing the top 10 GO term enrichment pathways from upregulated and downregulated DEGs in the DNE and NNE groups.

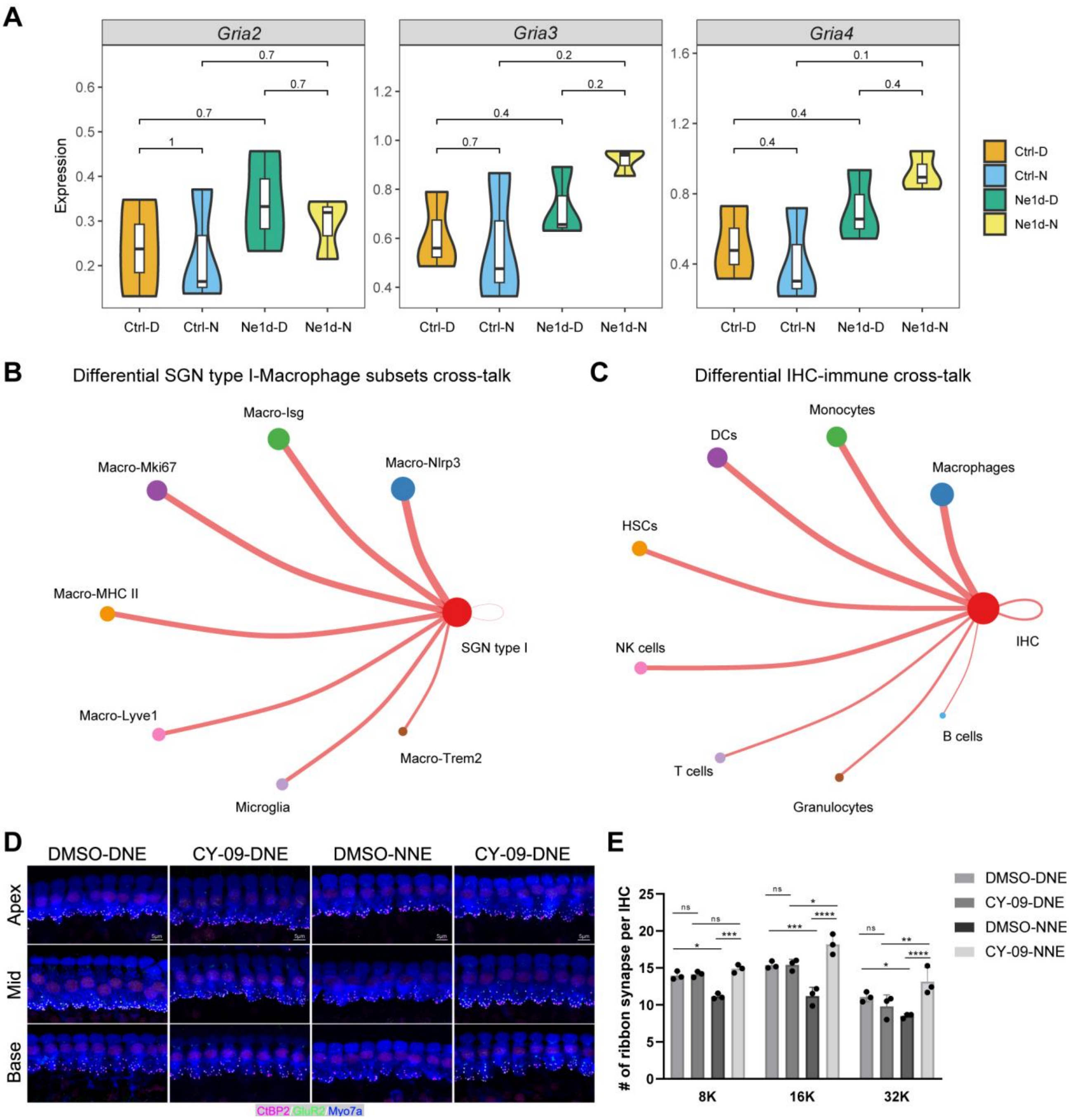

Fig. S6. Macrophage-driven immune interactions and non-immune cell vulnerability following NNE. **A** Violin plots showing the expression of *Gria2–4* genes in bulk RNA-seq data. **B** Ranked differential cross-talk analysis between SGN type I and macrophage subsets, with *Nlrp3*$^{+}$ macrophages ranking first among all ligand-receptor pairs. **C** Ranked differential cross-talk analysis between IHCs and immune cells, with macrophages ranking first among all ligand-receptor pairs. **D** Representative confocal images of IHCs co-labeled with the presynaptic marker CtBP2 (pink) and the postsynaptic

marker GluR2 (green) 14 days after DNE and NNE, comparing the CY-09-treated group and control group. Scale bars, 5 μm. **E** Numbers of ribbon synapses per IHC. Puncta co-labeled with CtBP2 and GluR2 are counted as ribbon synapses. The average number of synapses per IHC is calculated from 1–2 fields of view per frequency per mouse. Data are presented as the mean ± SD from 3 mice per group, replicated across three independent noise exposures (ns, no significant difference, $*P < 0.05$, $**P < 0.01$, $***P < 0.001$, $****P < 0.0001$, two-way ANOVA, Bonferroni *post hoc* test).

Supplemental Tables

Supplemental Table S1 Marker genes for immune and non-immune cells, related to Fig. 1.

Supplemental Table S2 Marker genes for immune and non-immune cell subsets, related to Figs 1 and 3.

Supplemental Table S3 DEGs for non-immune cell subsets, comparing DNE and NNE to control, related to Fig. 6.

Supplemental Table S4 Enrichment pathway analysis of SGN type 1 subsets, related to Fig. 6.
